# Effect of Surfactant Mixtures on the Evaporation Rate of Aqueous Sessile Droplets from Slightly Hydrophobic Substrates

**DOI:** 10.1021/acs.langmuir.5c03712

**Published:** 2025-09-08

**Authors:** Kristo Kotsi, Teng Dong, Takeshi Kobayashi, Alexander Moriarty, Ian McRobbie, Alberto Striolo, Panagiota Angeli

**Affiliations:** † ThAMeS Multiphase, Department of Chemical Engineering, 4919 University College London, Torrington Place, London WC1E 7JE, U.K.; ‡ Department of Chemical Engineering, University College London, Torrington Place, London WC1E 7JE, U.K.; § Innospec Ltd., Oil Sites Road, Ellesmere Port, Cheshire CH65 4EY, U.K.; ∥ School of Sustainable Chemical, Biological and Materials Engineering, Sarkeys Energy Center, The University of Oklahoma, Norman, Oklahoma 73019, United States

## Abstract

The evaporation of surfactant-laden sessile droplets has widespread applications in both natural and technological contexts. This study explores the evaporation of droplets containing a nonionic surfactant (tristyrylphenol ethoxylates (EOT)), an anionic surfactant (sodium benzenesulfonate with alkyl chain lengths of C_10_–C_13_ (NaDDBS)), and their mixtures at *n*_EOT_/*n*_NaDDBS_ mole ratios of 0.01, 0.1, 1, and 4, deposited on slightly hydrophobic silane-coated glass slides. The surfactants present significantly different critical micelle concentrations. In all cases studied, surfactants decreased the evaporation time compared to that of the pure water droplets. As the initial surfactant concentration increased, the evaporation time decreased. Interestingly, EOT-laden droplets exhibited longer evaporation times, despite EOT decreasing surface tension more than NaDDBS. For the EOT/NaDDBS mixtures, evaporation times fell between those of the surfactants alone, which was attributed to synergistic effects at the interface. The presence of surfactants tends to flatten the droplet, increase the surface area, and disrupt hydrogen bonds at the surface, which are likely to contribute to reducing the evaporation time; on the other hand, surfactant molecules tend to form layers at the surface, which hinder evaporation. The results were interpreted based on two distinct modes of evaporation, i.e., the constant contact radius and the constant contact angle; while both modes of evaporation were observed in all cases, the duration of the constant contact radius mode increased with surfactant concentration. As the concentration increased, deviations were observed between the results and predicted trends.

## Graphical abstract

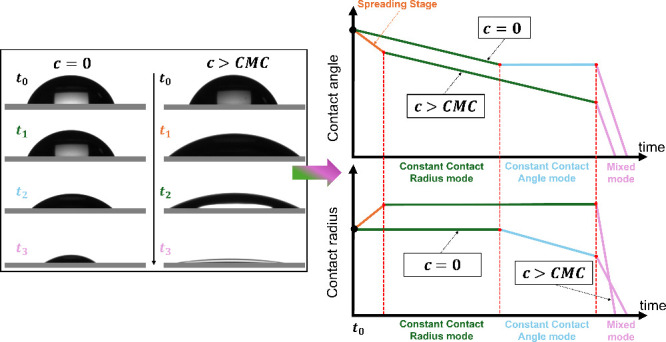

## Introduction

Droplet evaporation plays a significant role in climate and natural processes,^1^ including the survival of organisms^2^ and the water cycle.^3^ Droplet evaporation also affects industrial operations such as heat exchange,^4^ fire extinguishing,^5^ agricultural spraying,^6,7^ battery thermal management,^8^ inkjet printing,^9^ colloidal crystal growth for photonics,^10,11^ microseparation,^12^ DNA patterning,^13^ and disease diagnosis,^14^ among others. In industrial operations, droplet evaporation mainly occurs on solid substrates. The process is influenced by a multitude of factors, including surface temperature, humidity, mass transfer between liquid and vapor phases, and heat transfer among the substrate, liquid, and vapor phases.^15,16^ The inclusion of surfactants adds further complexity, as evaporation can be linked to surfactant self-assembly and self-organization,^17^ and the formation of patterns on the solid substrates due to their effect on the evaporation rate.^18^

The evaporation of pure liquid droplets can be divided into three modes for partial wetting of the substrate:^4,15,19–22^ (a) an initial pinned phase, where the contact radius remains constant but the contact angle decreases over time (constant contact radius (CCR) mode), (b) an unpinned phase, where the contact radius decreases while the contact angle does not change over time (constant contact angle (CCA) mode), and (c) a second unpinned phase, where both the contact angle and the contact radius rapidly decrease, until the droplet completely evaporates. A short spreading stage (SS) characterized by a rapid increase in contact radius and a swift decrease in droplet height upon deposition on the solid substrate has also been observed. The transitions between each mode are influenced by factors such as surface energy,^23^ droplet size,^24^ composition,^25^ surface roughness, and chemical heterogeneity.^26–28^

In many industrial processes, surfactants are added to droplets to alter their evaporation dynamic behavior^7^ or the wettability of the substrates^29^ with the evaporation process being overall influenced by the surfactant type and concentration, the solid substrate properties, and the environmental conditions. Surfactants influence evaporation because of two competing mechanisms: (a) by reducing the contact angle or increasing the contact area, thus changing the overall droplet profile and accelerating evaporation,^30–34^ according to Fick’s diffusion law or Maxwell–Stefan’s equations^35^ (for multicomponent systems) and (b) by forming layers at the interfaces that hinder evaporation.^25,36^ The literature remains inconclusive regarding which of these mechanisms influences the evaporation process the most, although the change in droplet profile seems to be the predominant factor.

Studies, like that of Kwieciński et al.,^31^ highlight the complexity of describing evaporation dynamics in surfactant-laden droplets, mainly because of challenges in precisely predicting the time-dependent surface tension, as noted by Esmaeli et al.^7^ However, various attempts have been made so far to understand this complex phenomenon. For instance, Birdi and Vu^37^ investigated sessile droplets containing 17 mM sodium dodecyl sulfate (SDS) on hydrophilic glass surfaces and found that the addition of surfactants reduces the contact angle and pins contact radius, overall promoting evaporation. Mohammadi et al.^23^ also reported an increased surface wettability, for water droplets with different surfactant types (anionic, cationic, and nonionic) deposited on superhydrophobic substrates. Additionally, Sefiane^30^ by depositing Triton X-100-laden water droplets on heated hydrophilic aluminum plates made similar observations; surfactants can enhance water evaporation by up to 75%, with the primary contributing factor reported to be the increase in the droplet contact area with the substrate. Finally, Aldhaleai and Tsai^38^ studied the evaporation of water droplets with didodecyldimethylammonium bromide (DDAB) on superhydrophobic and patterned polydimethylsiloxane solid surfaces and found a contact angle-dependent evaporation rate described by a universal curve and influenced by surfactant concentration.

However, Doganci et al.^25^ found that adding SDS to water droplets had a minimal impact on the droplet evaporation rate when deposited on hydrophobic surfaces; a slight reduction was mentioned only when the concentration notably exceeded the critical micelle concentration (CMC), which was believed to result from the formation of surfactant barriers at the water–air interface, limiting the available surface area for evaporation. Fainerman at al.^36^ also reported that adsorbed layers of ordinary surfactants, such as SDS and C_14_EO_8_ (an ethoxylated surfactant), do not significantly affect the evaporation rate, but in this case, droplets were pendant. However, on hydrophobic rice leaf surfaces^33^ and at concentrations equal to or above the CMC, it was observed that evaporation is faster, compared to lower concentrations, where a narrow thin air layer is trapped between the liquid–solid interface restricting vapor diffusion, a phenomenon also observed with sessile pure droplets on superhydrophobic patterned surfaces.^39^ Lastly, other studies for droplets on hydrophilic cotton leaves^32^ showed that above the CMC, surfactant layers at the droplet boundaries slow evaporation.

Recently, Shi et al.^40^ studied the evaporation of aqueous droplets containing single nonionic (polyoxyethylene glycol monoalkyl ethers) or anionic (SDS) surfactants on plasma-treated hydrophilic glass slides, by using interferometry to track the three-phase contact line. For the SDS-laden droplets, researchers^40^ found dried surfactant residues, after evaporation. In contrast, the drying behavior of the nonionic droplets suggested phase separation during evaporation. In particular, it was assumed that at the contact line, the solution separates into a water-rich phase that retracts, forming a surfactant-rich rim of higher viscosity, which facilitates contact line pinning, a behavior also reported by Kwieciński et al.^31^ for SDS-laden droplets on hydrophobic substrates.

In practical applications, mixtures of multiple surfactants are introduced into the liquids. Li et al.^41^ explored the impact of a mixed anionic surfactant system, consisting of an amphiphilic sodium alginate derivative (Ugi-Alg) and alkyl glycoside (APG), on the wettability of pesticide oil-in-water emulsions, on banana leaves. The surfactant system was found to considerably improve the wettability of the leaf surfaces, with observations linked to the decreasing surface tension at concentrations below and above the CMC, where unsaturated or saturated interfacial films form. However, no further evidence was provided by the researchers^41^ to support their observations. In addition, the combined surfactants had similar properties, while in everyday products, mixtures of surfactants of considerably different physical and chemical properties are often encountered.

To fill the knowledge gap in the literature, the aim of this work is to fundamentally investigate whether changes in the droplet profile or the formation of surfactant interfacial layers mainly influences the evaporation process of aqueous droplets laden with single surfactants. The study also explores the evaporation of mixed surfactant-laden aqueous droplets, a complex area rarely studied in the existing literature, and evaluates the relative effects of the droplet profile or surface composition as determined by the regular solution theory. Two surfactants, one nonionic and one anionic, often used in agriculture, are chosen because of their pronounced differences in CMC, ionic nature, and molecular weight. Our prior results^42,43^ show that the nonionic surfactant tristyrylphenol ethoxylates (EOT) is more effective in decreasing the surface tension of deionized (DI) water than the anionic surfactant sodium benzenesulfonate (NaDDBS) and that synergistic effects between the two surfactants occur, because of the solvation of Na^+^ ions at the interface by the nonionic surfactant.

## Evaporation of Sessile Droplets on Hydrophobic Substrates

To describe the evaporation of sessile droplets, it is key to quantify the rate of the change in volume (*V*) over time (*t*), d*V*/d*t*. Assuming the two-dimensional spherical cap geometry shown in Figure S1 and diffusion-controlled evaporation, theoretical models have been developed by Picknett and Bexon,^44^ Rowan et al.,^45^ and Bourgés-Monnier and Shanahan.^46^ These models are summarized in Table 1 and further detailed in the Supporting Information, where the corresponding equations are also listed (eqs S-5–S-9).

**Table 1:** Evaporation Models for Pure Liquid Droplets on Solid Substrates

model	remarks
P&B: 1 $\frac{dV}{dt}=-\frac{2\pi D}{\rho_{l}}\left(c_{\mathrm{SC}}-c_{\infty }\right)C$ ^44^	The model determined the capacitance of an equiconvex lens and is accurate for droplets with masses from 1 pg to 40 mg.
	Constant contact radius and constant contact angle modes were studied.
R: 2 $\frac{dV}{dt}=-\frac{2\pi R_{S}D}{\rho_{l}}\left(c_{\mathrm{SC}}-c_{\infty }\right)\left(1-\cos\;\theta \right)$ ^45^	A constant, radially outward concentration gradient was assumed, with a linear relation between contact angle *θ* and evaporation time *t* for 30° < *θ* < 90°.
	The constant contact radius mode was studied.
B&S: 3 $\frac{dV}{dt}=\frac{2\pi R_{S}D}{\rho_{l}}\left(c_{\mathrm{SC}}-c_{\infty }\right)\frac{\cos\;\theta }{\ln\left(1-\cos\theta \right)}$ ^46^	Evaporation was assumed to be radial within a specific region defined by two coaxial spherical caps of different radii.
	The constant contact radius mode was studied.

In the equations shown in Table 1, *ρ*_l_ is the liquid density, *R*_S_ is the radius of the sphere from which the spherical cap is obtained, as shown in Figure S1, *c*_SC_ is the concentration of the water vapor around the spherical cap, *c*_∞_ is the concentration of the vapor at infinite distance from the droplet, *D* is the diffusivity of the vapor to the surrounding air, and *C* is the capacitance of an isolated equiconvex lens of the shape and size of the droplet.

Employing basic geometric analysis, the radius of the sphere (*R*_S_) can be replaced by a term involving the volume of the sessile droplet (*V*) (see eq S-3). Thus, eqs 1–3 in Table 1 can be expressed in the following generalized form

4 $$\frac{dV}{dt}=-a\frac{V^{1/3}}{{\left(2-3\;\cos\;\theta +\mathrm{co}s^{3}\;\theta \right)}^{1/3}}g\left(\theta \right)$$

where *a* = $\frac{4\pi^{2/3}\times 3^{1/3}D}{\rho_{l}}\left(c_{\mathrm{sc}}-c_{\infty }\right)$ is considered constant and *g*(*θ*) is a function of the contact angle that for the different models is equal to^44–46^

5 $$g{\left(\theta \right)}_{\mathrm{P\&B}}=\frac{C}{2R_{S}}$$

6 $$g{\left(\theta \right)}_{R}=\frac{1-\cos\theta }{2}$$

7 $$g{\left(\theta \right)}_{\mathrm{B\&S}}=-\frac{\cos\theta }{2\;\ln\left(1-\cos\theta \right)}$$

The vapor concentration around the droplet surface, in eq 4, is given by

8 $$c_{SC}=\frac{P_{v}M_{w}}{RT}$$

where *M*_W_ = 18 g/mol is the vapor molecular weight, *R* = 8.314 × 10^6^ cm^3^ Pa K^–1^ mol^–1^ is the ideal gas constant, *T* = 294.15 K is room temperature, and *P*_V_ = 2339.3 Pa is the vapor pressure at room temperature.^47^

If we consider that the variable

9 $$\kappa \left(\theta \right)=\frac{g\left(\theta \right)}{{\left(2-3\;\cos\;\theta +\cos^{3}\;\theta \right)}^{1/3}}$$

remains time-independent during the whole evaporation process, eq 4 can be integrated with boundary conditions *t* → 0, *V* → *V*_max_ and *t* → *t*_evap_, *V* → *V*_*t*_evap__, which after normalization results in

10 $${\left(\frac{V_{t_{\mathrm{evap}}}}{V_{\max}}\right)}^{2/3}={\left(\frac{1}{V_{\max}}\right)}^{2/3}\left({V_{\max}}^{2/3}-\frac{2}{3}a\kappa \left(\theta \right)t_{\mathrm{evap}}\right)$$

Equation 9 is used in eq 10, which is utilized below to fit our experimental data. The modeling analysis in the work of Semenov et al.^48^ has shown that the evolution of the contact angle in the CCR mode and the evolution of the contact radius in the CCA mode are not affected by the presence of surfactants, and the (*V*/*V*_max_)^2/3^ ∼ *t* law still applies in surfactant systems.

## Experimental Materials and Procedures

### Materials

A nonionic surfactant (98 wt % tristyrylphenol ethoxylates (EOT); MW = 1122 g/mol) and an anionic surfactant (25 wt % sodium benzenesulfonic acid with C_10_–C_13_ alkyl chain lengths (NaDDBS); MW = 348 g/mol), both provided from Innospec, were used. Representative structures are shown in Figure S2. EOT mostly incorporates 16 ethoxylate groups, while NaDDBS molecules mostly have 12-carbon alkyl chains. DI water (resistivity ρ = 18.2 MΩ cm; conductivity κ = 0.055 μS/cm) was used as the base liquid for the droplet-generating solutions; the preparation process has already been described in our previous work.^42^ For the mixtures, *n*_EOT_/*n*_NaDDBS_ mole ratios of 0.01, 0.1, 1, and 4 were considered.

The surface tension was measured at ∼21 °C using a force balance K100C tensiometer (Krüss Scientific, Germany) equipped with a Wilhelmy plate. All concentrations were tested three times, and the average equilibrium values with standard deviation error bars are shown in Figure 1.

**Figure 1:**
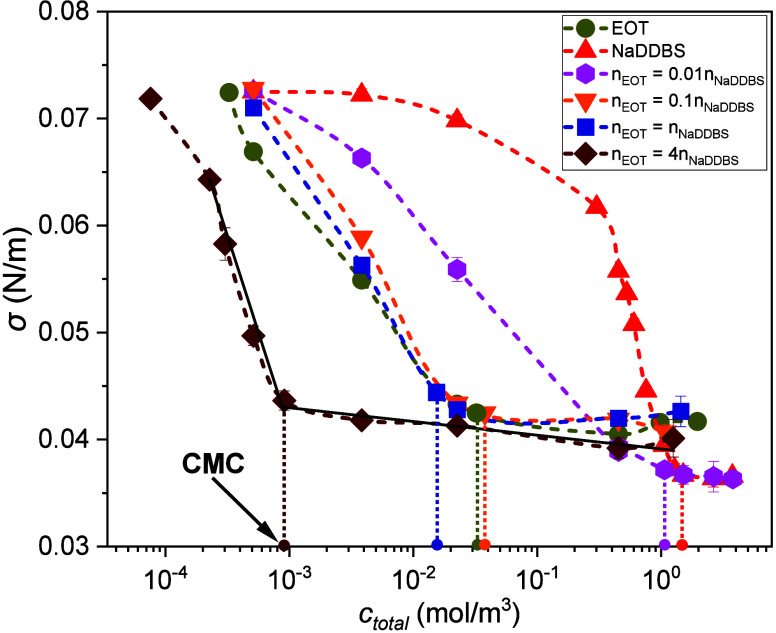
Equilibrium surface tension for all of the droplet-generating solutions. Dashed lines are guides for the eye, while dotted vertical lines indicate the CMC for each case. In general, symbols are larger than the uncertainty. The error bars indicate standard deviations.

To perform the measurements, the sample vessel was filled with 20 mL of a surfactant-laden aqueous solution. To eliminate the effect of impurities, both the plate and the vessel were initially rinsed with DI water and then with methanol (≥99%) and finally flame-cleaned using a Bunsen burner. From the surface tension data (Figure 1), CMCs were extracted for the single surfactants and surfactant mixtures, as the intersection point between the linearly decreasing premicellar region and the line passing through the plateau in the postmicellar region,^42^ indicatively shown in Figure 1 for the *n*_EOT_/*n*_NaDDBS_ = 4 mixed case. CMCs are summarized in Table 2.

**Table 2:** CMC Values for the Surfactants Alone and Their Mixtures^42^

case	CMC (mol/m^3^)	case	CMC (mol/m^3^)
EOT	3.68 × 10^–2^	*n*_EOT_/*n*_NaDDBS_ = 0.1	3.92 × 10^–2^
NaDDBS	1.28	*n*_EOT_/*n*_NaDDBS_ = 1	1.72 × 10^–2^
*n*_EOT_/*n*_NaDDBS_ = 0.01	1.06	*n*_EOT_/*n*_NaDDBS_ = 4	9.10 × 10^–4^

### Droplet Evaporation Measurements

The DSA100 drop shape analyzer (Krüss Scientific, Germany) was employed to measure the base radius and contact angle of the evaporating droplets, as shown in Figure 2. Before the measurements, the captured grayscale image is calibrated (pixels to millimeters) based on the outer diameter of the syringe needle, used to generate the droplet.

**Figure 2:**
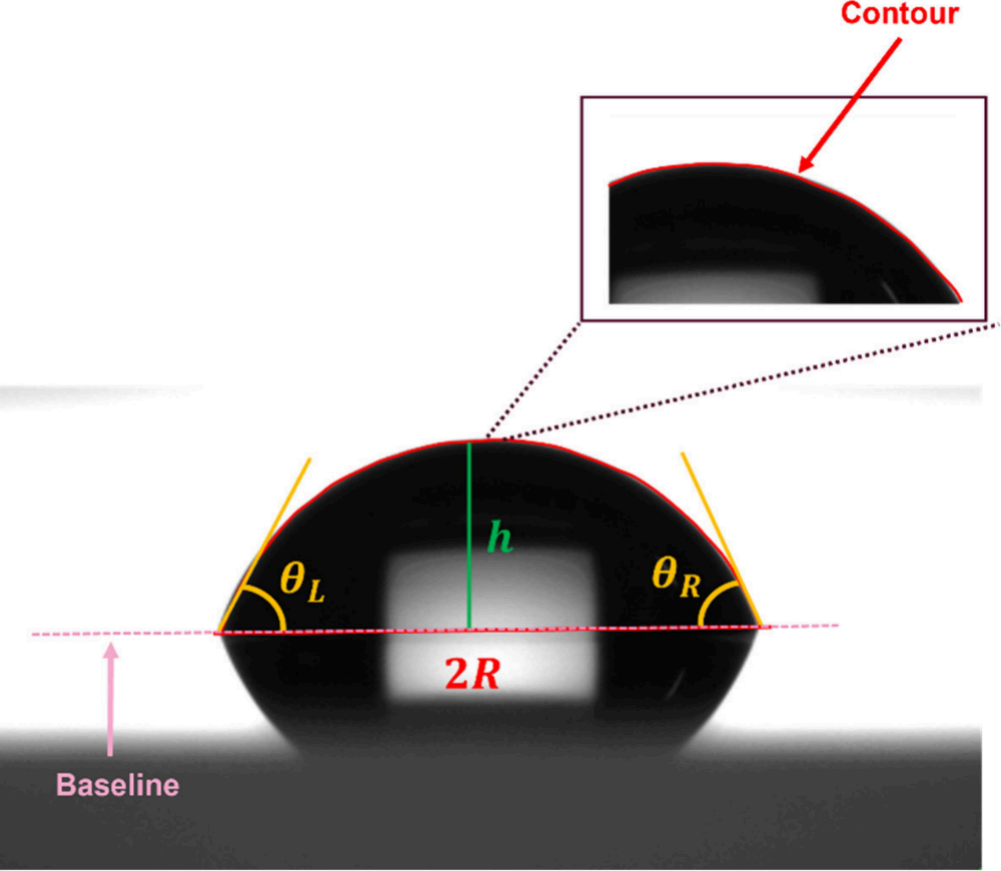
Contact angle and radius measurements for a sessile droplet on a solid substrate.

The digital image is numerically analyzed to detect the droplet contour (boundaries) and fit the geometrical model that best describes its shape. Contact angles are determined at the left and right droplet edges, where the contour intersects the baseline, and an average is taken to characterize surface wettability.

Droplets were deposited on silane-coated glass slides (Agilent Technologies, USA) and used as received. To quantify the adsorption of surfactants on this solid substrate, quartz crystal microbalance measurements were conducted on silane-coated silica sensors (Biolin Scientific AB, Sweden; see the Supporting Information). Silane coating offers a surface modification that enhances the hydrophobicity of the glass substrate so that contact angles are not very small and can be easily detected. For pure DI water droplets, the average initial static contact angle was *θ* ∼ 70 ± 1.5°. Additionally, glass substrates offer a reflective surface, which facilitates more accurate detection of the three-phase contact line.

For the evaporation measurements, a droplet with a volume of ∼3.3 ± 0.3 μL was carefully deposited on the silane-coated glass slide. The droplet shape was monitored over time until it could no longer be accurately detected (when its volume was smaller than ∼1.1 ± 0.2 μL). For all of the cases studied, once the droplets were deposited on the substrate, their contact radius was *R* = 1.4 ± 0.1 mm. This gave a capillary length (eq S-1) between 1.9 and 2.7 mm and a Bond number (eq S-2) between 0.3 and 0.7, satisfying the conditions (*R* < λ and *Bo* < 1) that confirm that all droplets could be modeled by a spherical cap.^49,50^ During evaporation, the droplet volumes were calculated with MATLAB and matched those obtained from DSA100 (Figure S3). To confirm that droplets retained a spherical cap geometry in both constant contact radius and constant contact angle modes, MATLAB results were compared with the theoretical volumes from eqs S-3 and S-4, using DSA100-measured contact angles and radii. As indicatively shown in Figure S4 for pure DI water and EOT-laden droplets, the two methods agreed closely.

Contact angle values were captured every Δ*t* = 10 s, but for the sake of clarity, only every fourth point is shown in Figure 10 and Figure S9. Measurements were carried out at room temperature (∼21 °C) and a relative humidity (RH) of 35–40%, monitored with a humidity sensor placed near the droplet. To ensure that small changes in ambient humidity did not affect evaporation, the contact angle of a pure DI water droplet was measured over time at the beginning and end of each set of experiments, with the evaporation pattern for the test droplets not changing significantly; the variation in the measured evaporation times was within 3%.

## Results and Discussion

### Evaporation Time

The evaporation time is defined as the total time required for a droplet to completely evaporate from the moment it is deposited on a substrate. As one can see in Figure 3, compared to the pure DI water case, the presence of either single EOT or NaDDBS surfactants, as well as of their mixtures, decreases the evaporation time. In addition, as the total initial surfactant concentration increases, the evaporation time decreases. To interpret the results, both changes in droplet profile and surfactant adsorption at the water–air interface are considered for the single surfactants and their mixtures.

**Figure 3:**
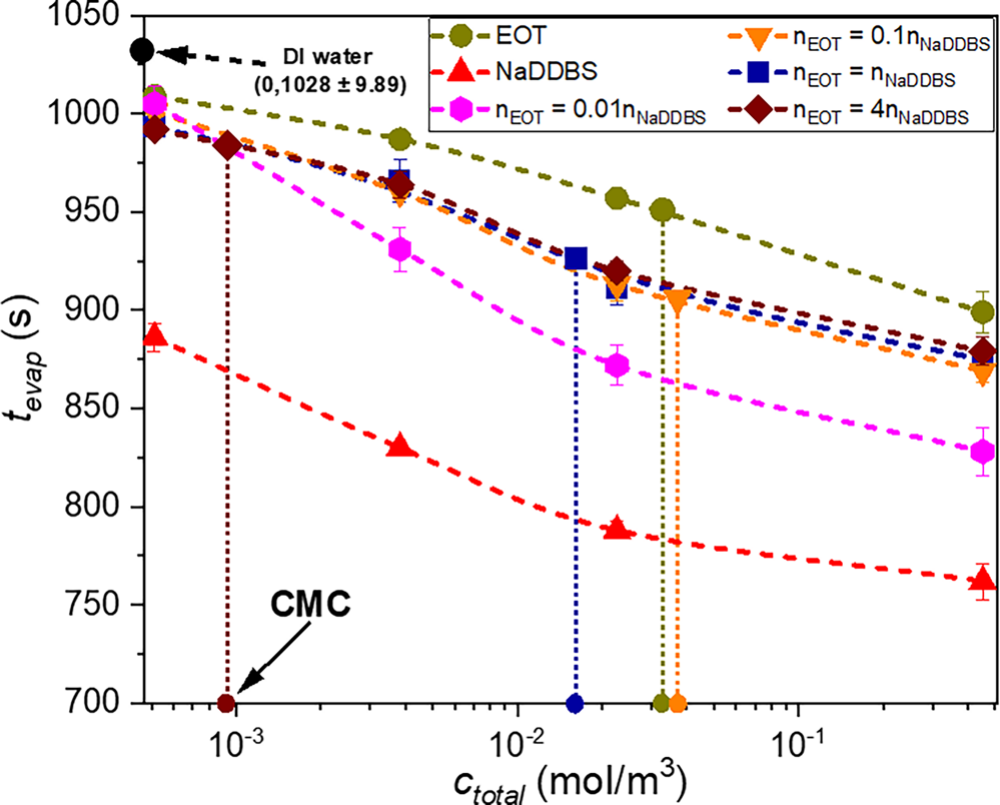
Evaporation times for the surfactant-laden droplets. Dashed lines are guides for the eye, while dotted vertical lines indicate CMCs estimated from Figure 1. Error bars represent the standard deviation of the three measurements. The symbols are generally larger than the error bars.

#### Single Surfactants

As shown in Figure 3, and compared to pure DI water droplets, the evaporation time of droplets containing the nonionic (EOT) surfactant decreases with an increase in EOT concentration. At concentrations below the CMC of EOT (CMC_EOT_ = 3.68 × 10^–2^ mol/m^3^), such as at *c*_total_ = 5.13 × 10^–4^ mol/m^3^ and *c*_total_ = 3.83 × 10^–3^ mol/m^3^, the decrease in evaporation time is rather small, i.e., *t*_evap_ = 1009 s (1.8% decrease) and *t*_evap_ = 987 s (4.0% decrease), respectively, and comparable to the pure DI water droplets (*t*_evap_ = 1028 s). At higher concentrations, the evaporation time decreases to 957 s (a 6.9% decrease) at *c*_total_ = 2.26 × 10^–2^ mol/m^3^ and to 899 s (a 12.5% decrease) at *c*_total_ = 4.52 × 10^–1^ mol/m^3^.

The decrease could be attributed to the decrease in surface tension with an increase in EOT concentration (Figure 1), which decreases the contact angle faster and flattens the droplet. A decrease in contact angle was indeed observed in Figure 10 and will be discussed further in the next section. However, the overall droplet shape as shown in Figure 4b does not change significantly compared to pure DI water droplets (Figure 4a). This is also confirmed from Figure 5, where the temporal evolution of the normalized droplet height to contact diameter ratio ((*h*/2*R*)_norm_) is comparable for both the DI water and EOT-laden droplets at *c*_total_ = 5.13 × 10^–4^ mol/m^3^; however, while DI water droplets remain symmetric, EOT-laden droplets exhibit a center shift after the constant contact angle mode of evaporation. Similar results were observed for *c*_total_ = 3.83 × 10^–3^ mol/m^3^, which are not shown here. These observations suggest that at low EOT concentrations the surfactant enhances local evaporation near the contact line by reducing the contact angle, without significantly affecting the droplet shape. In contrast, at the higher concentration of *c*_total_ = 2.26 × 10^–2^ mol/m^3^, the droplet shape flattens (see Figure 4d) and the (*h*/2*R*)_norm_ ratio in Figure 5 substantially decreases, leading to a rapid decrease in evaporation time. The same trend was found for *c*_total_ = 4.52 × 10^–1^ mol/m^3^ (not shown here for the sake of brevity).

**Figure 4:**
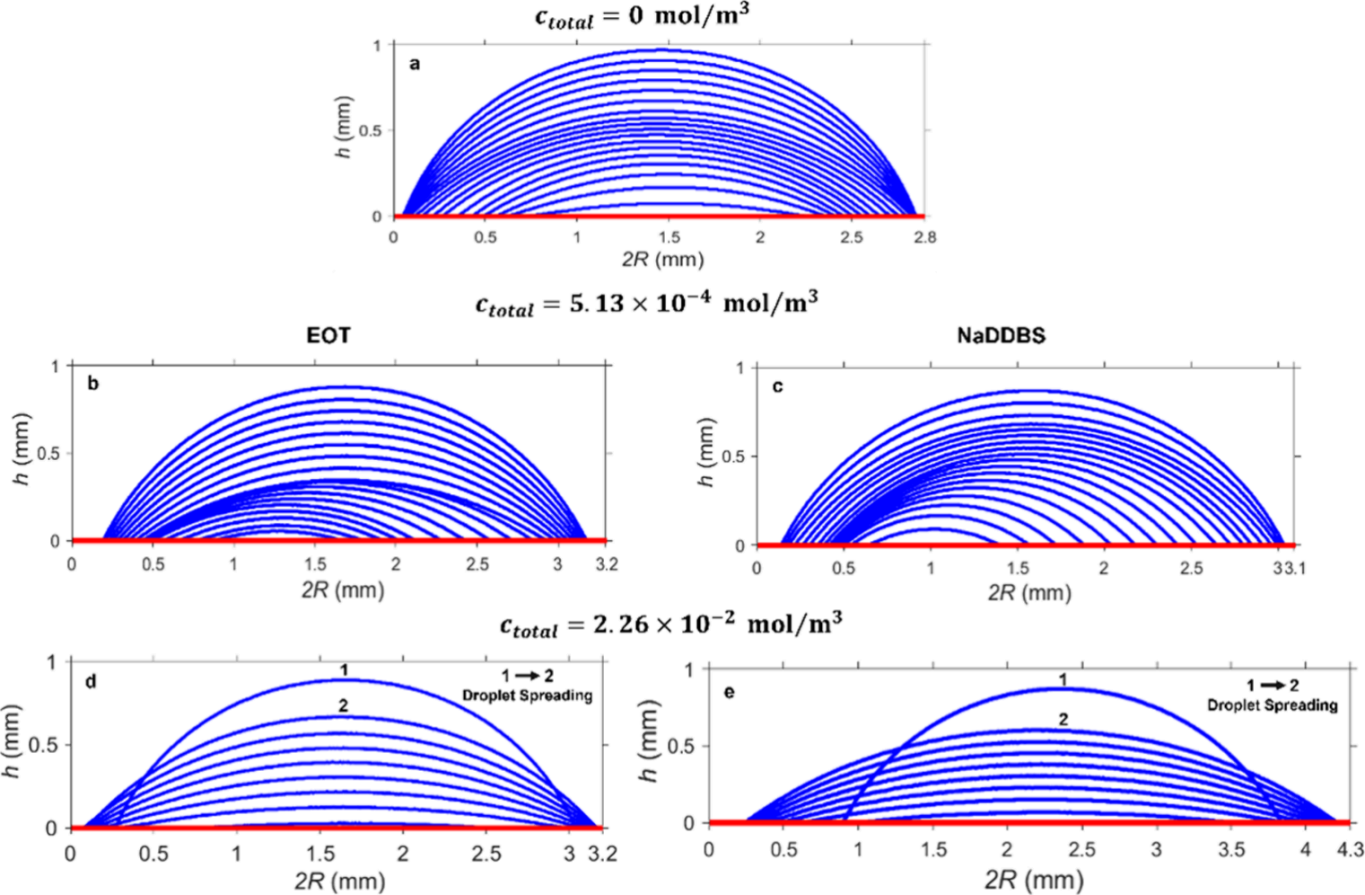
Evolution of the droplet profiles for (a) pure and (b–e) single surfactant-laden droplets.

**Figure 5:**
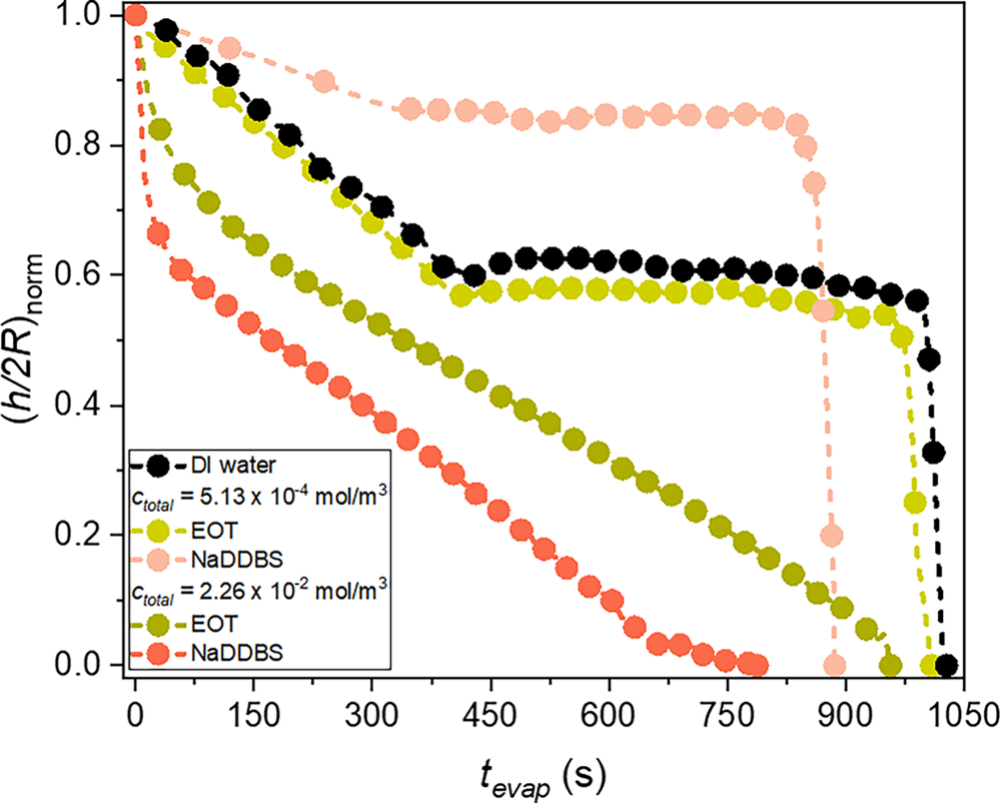
Temporal evolution of the normalized height/base diameter ratio for both pure and single surfactant-laden droplets.

As the EOT concentration within the droplet increases (Figure 6) so does its surface excess (Γ), as plotted in Figure 7. Temporal changes in surfactant concentration within the droplet were determined by dividing the initial number of moles, at *t*_evap_ = 0 s, by the decreasing droplet volume. The surface excess was estimated using the Langmuir isotherm, based on parameters (*Γ*_max_ and *K*_L_) obtained by fitting the Langmuir–Szyszkowski equation to the surface tension data, as described in our previous work.^42^ To allow for these calculations, we made three assumptions. First, based on the fast adsorption of EOT and NaDDBS at the water–air interface (within seconds^42^) and the low evaporation rates (∼10^–5^ cm^2^/s (Table 3 and Table S1)), evaporation was assumed to proceed through a series of quasi-equilibrium states. Second, it was assumed that surfactants do not evaporate, and thus, their total moles in the droplet remain constant. Finally, quartz crystal microbalance measurements (eqs S-10–S-12 and Figures S5 and S6) showed that the amount of surfactant adsorbed at the solid interface, at the highest concentration considered, was negligible compared to the amount of surfactant within the droplets; to simplify the analysis, this adsorption was neglected.

**Figure 6:**
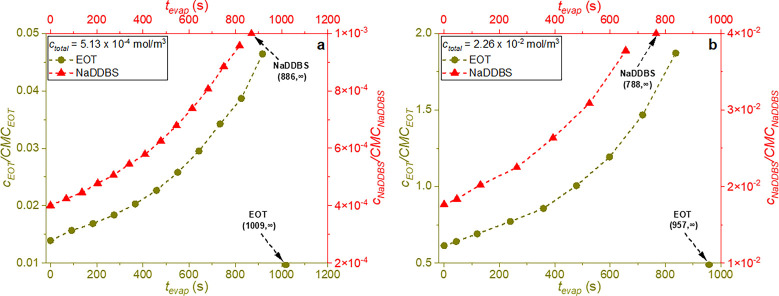
*c*/CMC evolution for the single EOT and NaDDBS cases, at (a) low and (b) high total initial surfactant concentrations. Lines are guides for the eye, and infinity symbols denote the surfactant concentration after complete droplet evaporation.

**Figure 7:**
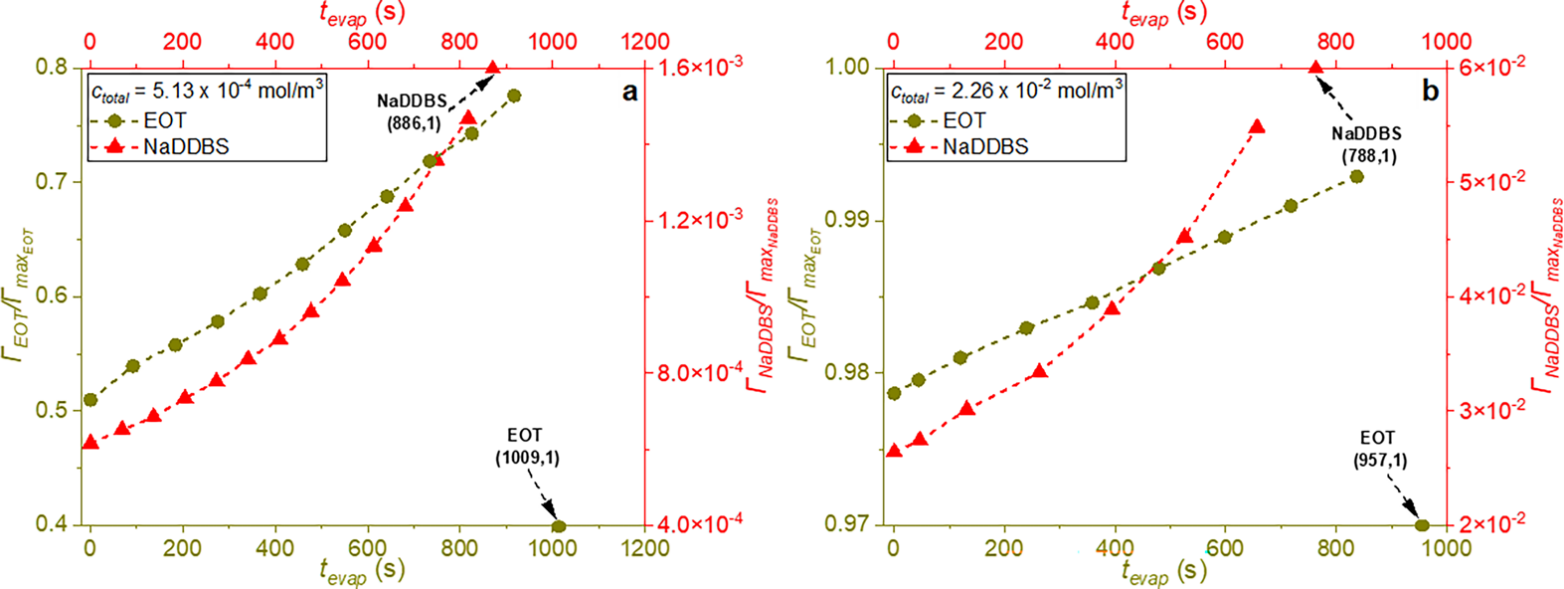
Surface coverage evolution for single EOT and NaDDBS cases, at (a) low and (b) high total initial surfactant concentrations. Lines are guides for the eye. Symbols with arrows indicate the surfactant surface concentration after complete droplet evaporation.

**Table 3:** Evaporation Rates for DI Water and EOT-Laden Droplets

EOT, Nonionic				
	ακ(θ) (× 10^–5^ cm^2^/s)			
*c*_EOT_ (mol/m^3^)	ακ(θ)_*E*_	ακ(θ)_*P&B*_	ακ(θ)_*R*_	ακ(θ)_*B&S*_
0	1.60	2.07	1.20	2.14
5.13 × 10^–4^	1.78	2.20	1.34	2.36
3.83 × 10^–3^	1.99	2.32		2.65
2.26 × 10^–2^	2.00	2.53		
4.52 × 10^–1^	2.03	2.77		

Figure 6a shows that the EOT concentration in the droplet gradually increases over time. At the same time, the EOT surface excess at the water–air interface increases linearly, with surfactant molecules almost covering the interface within *t*_evap_ ∼ 900 s at concentrations both below and above its CMC (*c*_total_ = 5.13 × 10^–4^ mol/m^3^, and *c*_total_ = 2.26 × 10^–2^ mol/m^3^ (shown in panels a and b, respectively, of Figure 7)). While it is possible that these surfactants form layers that act as barriers, slowing water evaporation, layer formation does not seem to significantly hinder evaporation, as the evaporation times shown in Figure 3 keep decreasing with an increase in surfactant concentration.

To shed light on the potential mechanisms, molecular dynamics simulations were conducted using GROMACS 2021.5 ^51–53^ (details in the Supporting Information). The results revealed that EOT molecules adsorb at the water–air interface, displacing surface water molecules from the interface (Figure S7a). The surface density of both water molecules and hydrogen bonds decreases, the latter by more than 30% (see Figure 8a) upon the addition of EOT. Livingstone et al.^54^ showed that the vibrational frequency of water molecules at the water–air interface, influenced by hydrogen bonding, is higher when the molecules are in direct contact with surfactants. As reported by Tummala et al.,^55^ this can result in shorter lifetimes for the surface waters, making it easier for them to escape, potentially decreasing evaporation times.

**Figure 8:**
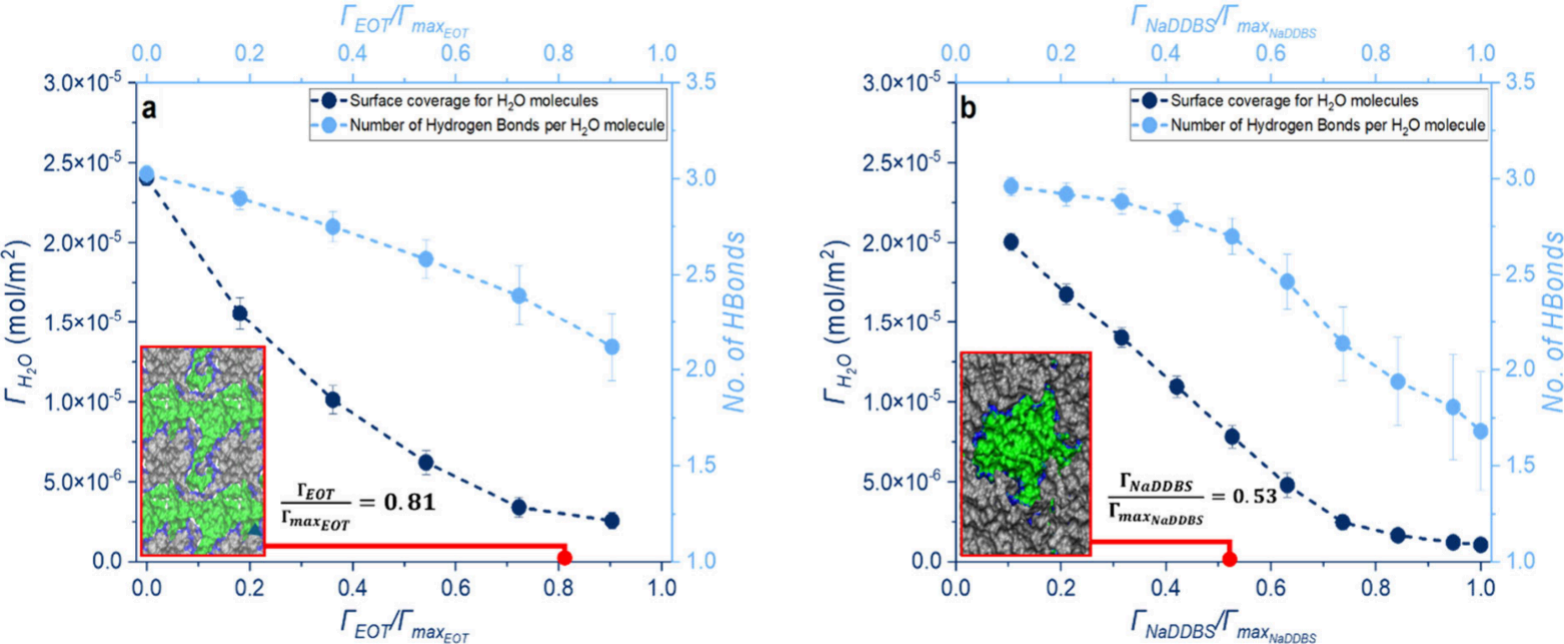
H_2_O surface coverage and hydrogen bonds over the (a) EOT and (b) NaDDBS surface excess. Insets are top views of the distorted water surface where gray molecules are surfactants; blue and green indicate nearby and distant water molecules, respectively.

Compared to EOT, and as shown in Figure 3, NaDDBS significantly reduces the evaporation time when present in DI water droplets even at concentrations much lower than its CMC, e.g., *t*_evap_ = 886 s (13.8% reduction) at *c*_total_ = 5.13 × 10^–4^ mol/m^3^. As the total NaDDBS initial concentration in the droplet increases, the evaporation time further decreases. At the higher concentrations tested, e.g., at *c*_total_ = 2.26 × 10^–2^ mol/m^3^, the NaDDBS droplets are flatter than those containing EOT (see Figure 5), which can explain the shorter evaporation times observed for NaDDBS than for EOT (Figure 3). At the lowest concentration tested, *c*_total_ = 5.13 × 10^–4^ mol/m^3^, droplets containing either surfactant have similar profiles once deposited on the substrate. However, the EOT-laden droplets exhibit a flatter shape compared to NaDDBS (Figures 4 and 5), which is expected since EOT reduces surface tension more than NaDDBS (Figure 1). Consequently, EOT droplets would be expected to evaporate faster, yet in Figure 3, the opposite is observed.

This discrepancy may be attributed to the adsorption dynamics of NaDDBS. As shown in Figure 6, the concentration of NaDDBS increases much faster than that of EOT for both low and high total initial concentrations, but its maximum concentration remains well below its CMC, reaching *c*_NaDDBS_/CMC_NaDDBS_ ∼ 3.78 × 10^–2^ when *c*_total_ = 2.26 × 10^–2^ mol/m^3^ (Figure 6b). As a result, in Figure 7, the NaDDBS surface excess increases more slowly, reaching a significantly lower value compared to EOT. It is worth noting that, in the presence of NaDDBS, hydrogen bonding decreases by approximately 50% at its saturated concentration (Figure 8b), though experimentally the NaDDBS surface excess only reaches a maximum of ∼0.06Γ_max_NaDDBS__ when *c*_total_ = 2.26 × 10^–2^ mol/m^3^ (Figure 7b). The sparse packing of NaDDBS molecules at the water–air interface (Figure S7b) likely explains the faster evaporation of the NaDDBS-laden droplets, despite the changes in droplet profiles. The thicknesses of the EOT and NaDDBS surface layers, under saturated conditions, are shown in Figure S8.

Compared to pure DI water droplets, NaDDBS-laden ones have a lower (*h*/2*R*)_norm_ ratio (Figure 5) and a flatter shape (see Figure 4e) at *c*_total_ = 2.26 × 10^–2^ mol/m^3^, which could explain the lower evaporation times when the surfactant is present. Similar results, not shown for the sake of brevity, were also obtained for *c*_total_ = 4.52 × 10^–1^ mol/m^3^. At the low concentration of *c*_total_ = 5.13 × 10^–4^ mol/m^3^, though (or of *c*_total_ = 3.83 × 10^–3^ mol/m^3^ (not shown here)), the ratio (*h*/2*R*)_norm_ is higher for NaDDBS than for DI water, which suggests a longer evaporation time. Additionally, the packing of NaDDBS molecules at the droplet surface could further hinder evaporation. However, as shown in Figure 3, NaDDBS droplets exhibit a shorter evaporation time. This discrepancy may be attributed to the reduction in hydrogen bonding at the interface caused by the NaDDBS molecules, especially at the end of evaporation (Figure 8b). In addition, Marangoni flows^21^ may establish which can influence evaporation; a more detailed investigation is required to uncover the underlying mechanisms.

#### Surfactant Mixtures

For the *n*_EOT_/*n*_NaDDBS_ = 0.01, 0.1, 1, and 4 droplets, at *c*_total_ = 5.13 × 10^–4^ mol/m^3^, evaporation times slightly decrease by approximately 3% across all cases, compared to pure DI water, despite the varying droplet profiles shown in Figure 9a. Therefore, at this concentration, the change in droplet shape due to the surfactants does not significantly impact the evaporation times. As the surfactant concentration further increases, the evaporation times decrease, and when *n*_EOT_/*n*_NaDDBS_ = 0.1, 1, and 4, evaporation times are comparable, and higher than those obtained for *n*_EOT_/*n*_NaDDBS_ = 0.01. Indeed, droplet profiles in Figure 9b–d confirm flatter droplets for *n*_EOT_/*n*_NaDDBS_ = 0.01, which accounts for the lower evaporation times in Figure 3, while the similar (*h*/2*R*)_norm_ ratios for the *n*_EOT_/*n*_NaDDBS_ = 0.1, 1, and 4 cases explain the small differences between their evaporation times. This suggests that the effects of surfactants and their mixtures on the evaporation time are not monotonic.

**Figure 9:**
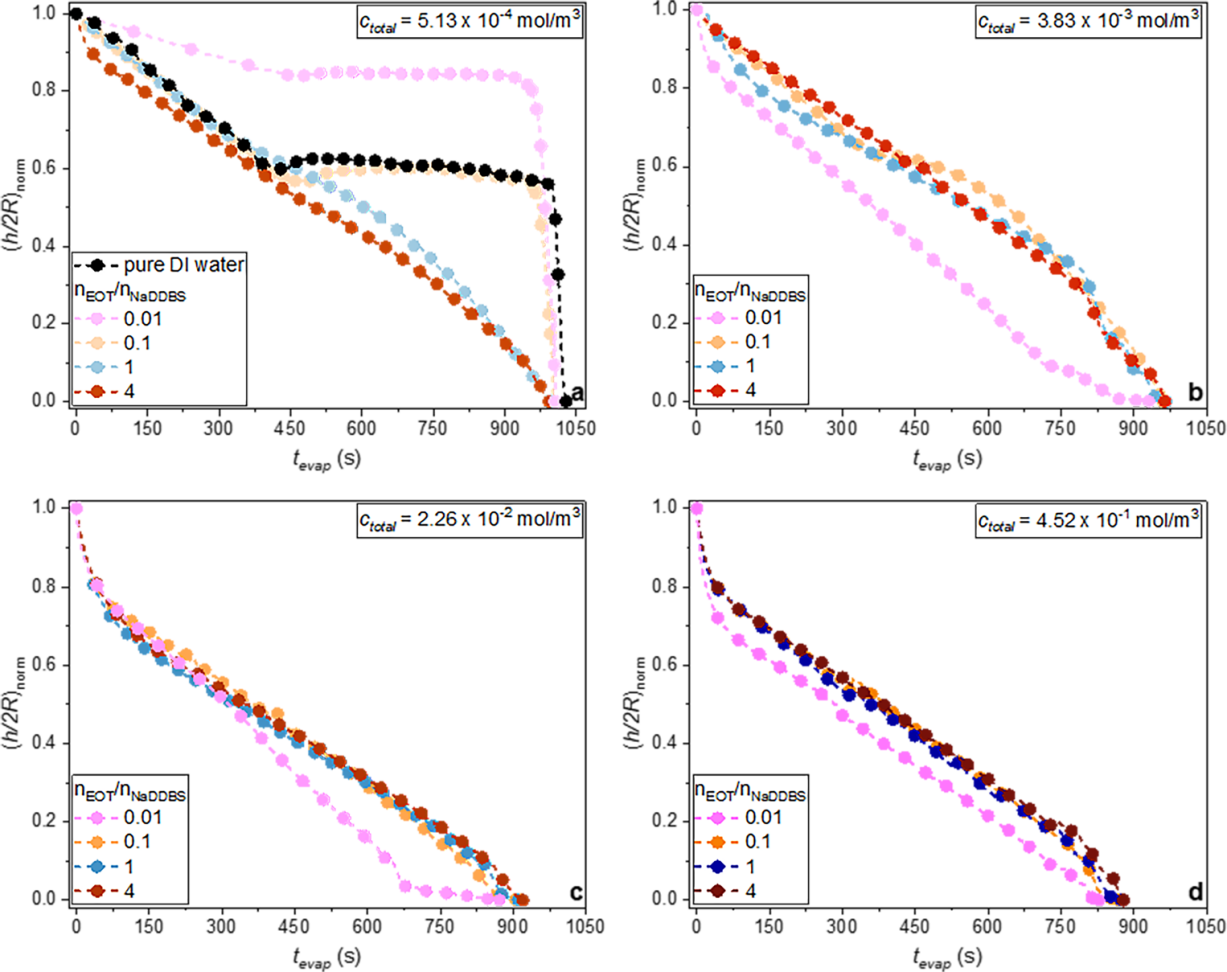
Temporal evolution of the normalized height/base diameter ratio for the mixed laden droplets, at *c*_total_ = 5.13 × 10^–4^, 3.83 × 10^–3^, 2.26 × 10^–2^, and 4.52 × 10^–1^ mol/m^3^ (panels a–d, respectively).

In addition, Figure 3 reveals that the addition of EOT to NaDDBS increases the evaporation times of the mixed laden droplets to values closer to those of the EOT-laden droplets. To interpret these results, we refer to our previous work^42,43^ on the synergistic effects between the nonionic (EOT) and anionic (NaDDBS) surfactants on surface tension. These synergistic effects increase as the EOT/NaDDBS mole ratio increases. By applying the regular solution theory, as proposed by Rosen and Hua,^56^ our prior analysis^42^ showed that, even at low mole ratios, i.e., *n*_EOT_/*n*_NaDDBS_ = 0.01, EOT dominates at the water surface ( $\overset{®}{X_{\mathrm{EOT}}\;}=0.505>\overset{®}{X_{\mathrm{NaDDBS}}\;}=0.495$ ), where $\overset{®}{X_{\mathrm{EOT}}\;}$ and $\overset{®}{X_{\mathrm{NaDDBS}}\;}$ are the average mole fractions of the nonionic (EOT) and anionic (NaDDBS) surfactants, respectively, at the water–air interface, as calculated in our previous work.^42^ For *n*_EOT_/*n*_NaDDBS_ = 0.1 and 4, the EOT mole fraction at the interface further increases to $\overset{®}{X_{\mathrm{EOT}}\;}=0.643$ , reaching $\overset{®}{X_{\mathrm{EOT}}\;}=0.806$ at *n*_EOT_/*n*_NaDDBS_ = 1; this could explain why the evaporation times for these ratios in Figure 3 are closer to those of single EOT. In the mixtures, EOT molecules preferably adsorb at the water surface, with a higher adsorption/desorption rate ratio (*K*_L_EOT__ = 2028.5 m^3^/mol) than NaDDBS (*K*_L_NaDDBS__ = 1.20 m^3^/mol), and tightly pack with NaDDBS molecules due to synergism.^42^ Overall, the results show that the distribution of surfactants at the interface, combined with the disruption of the hydrogen bond network, affects the evaporation times.

### Modes of Evaporation

An analysis of the profiles in Figures 5 and 9c,d reveals that droplets spread when *c*_total_ ≥ 2.26 × 10^–2^ mol/m^3^, for all cases studied, and when *c*_total_ ≥ 3.83 × 10^–3^ mol/m^3^ for mixtures with *n*_EOT_/*n*_NaDDBS_ = 1 and 4 (Figure 9b). During spreading, the droplet shape flattens, accompanied by a rapid decrease in droplet height and an increase in the contact radius. For instance, in Figure 5, NaDDBS-laden droplets at *c*_NaDDBS_ = 2.26 × 10^–2^ mol/m^3^ spread such that the (*h*/2*R*)_norm_ ratio abruptly decreases to 0.67. This phenomenon is not observed for pure DI water or for the lowest surfactant concentrations, as in these cases, a higher surface tension resists spreading and promotes retraction after droplet deposition on the solid surface. For example, as shown in Figure 4c, when *c*_NaDDBS_ = 5.13 × 10^–4^ mol/m^3^, only the NaDDBS-laden droplet height decreases, while its contact radius remains constant immediately after deposition.

Once spreading is complete, a pinning of the contact line is observed, and at high concentrations, the evaporation happens in the CCR mode with a continually decreasing (*h*/2*R*)_norm_ ratio as illustrated in Figure 5 for *c*_total_ = 2.26 × 10^–2^ mol/m^3^ and in Figure 9b–d for *c*_total_ ≥ 3.83 × 10^–3^ mol/m^3^. At the low concentration of *c*_total_ = 5.13 × 10^–4^ mol/m^3^, the contact line eventually retracts, leading to a transition to the CCA mode, where (*h*/2*R*)_norm_ remains almost constant, in Figures 5 and 9a for *n*_EOT_/*n*_NaDDBS_ = 0.01 and 0.1.

### Spreading Stage

In Figures 10 and 11, the temporal evolution of the contact angle and the contact radius, respectively, during evaporation for the single surfactants and surfactant mixture cases is presented. Here only the first (CCR mode) and second (CCA mode) stages of evaporation are reported, as they primarily contribute to the overall evaporation time, while the driving forces and kinetics of the third stage are more complex to analyze,^15^ probably due to its short duration.

**Figure 10:**
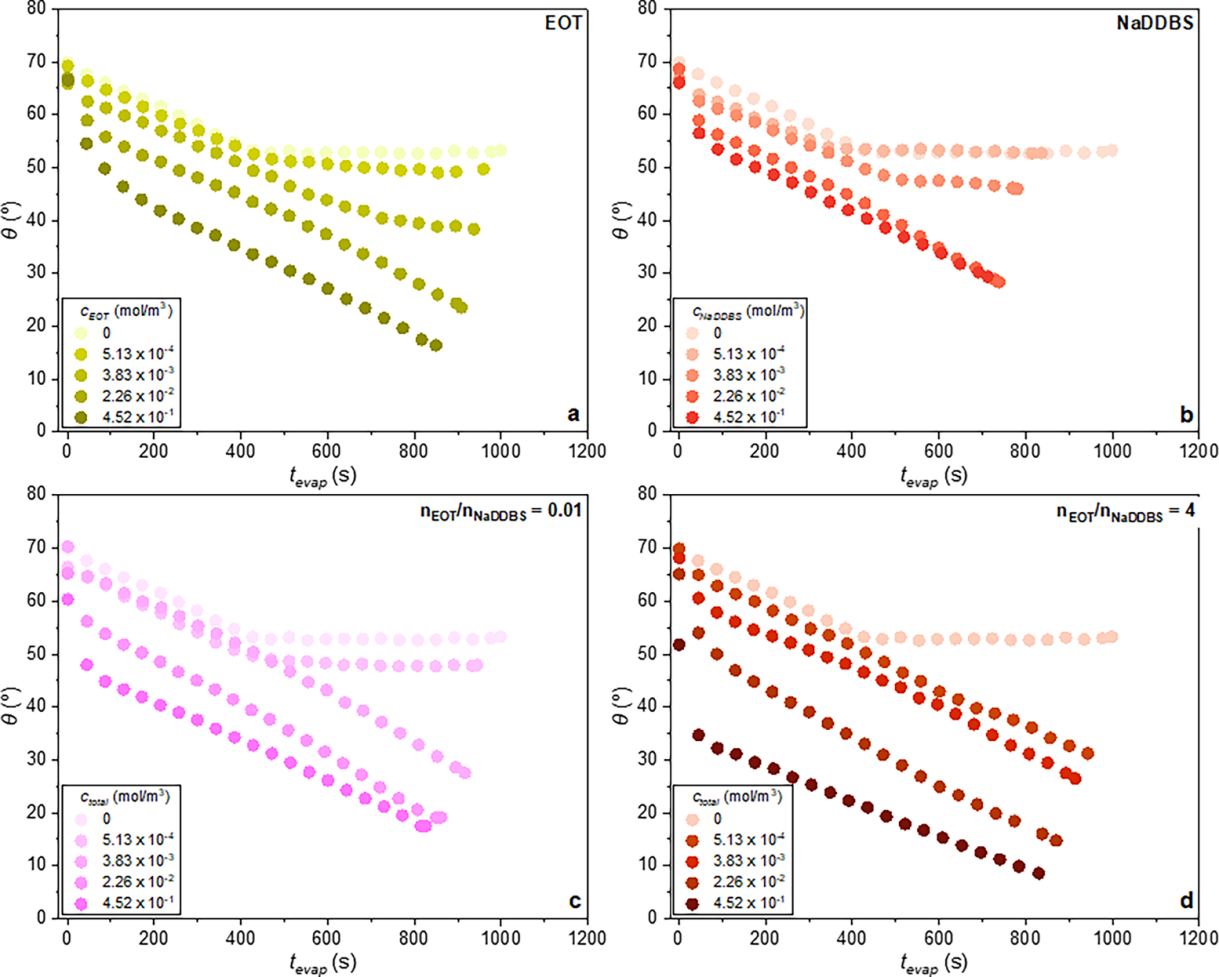
Evolution of the contact angle for the (a and b) single and (c and d) mixed surfactant-laden droplets.

**Figure 11:**
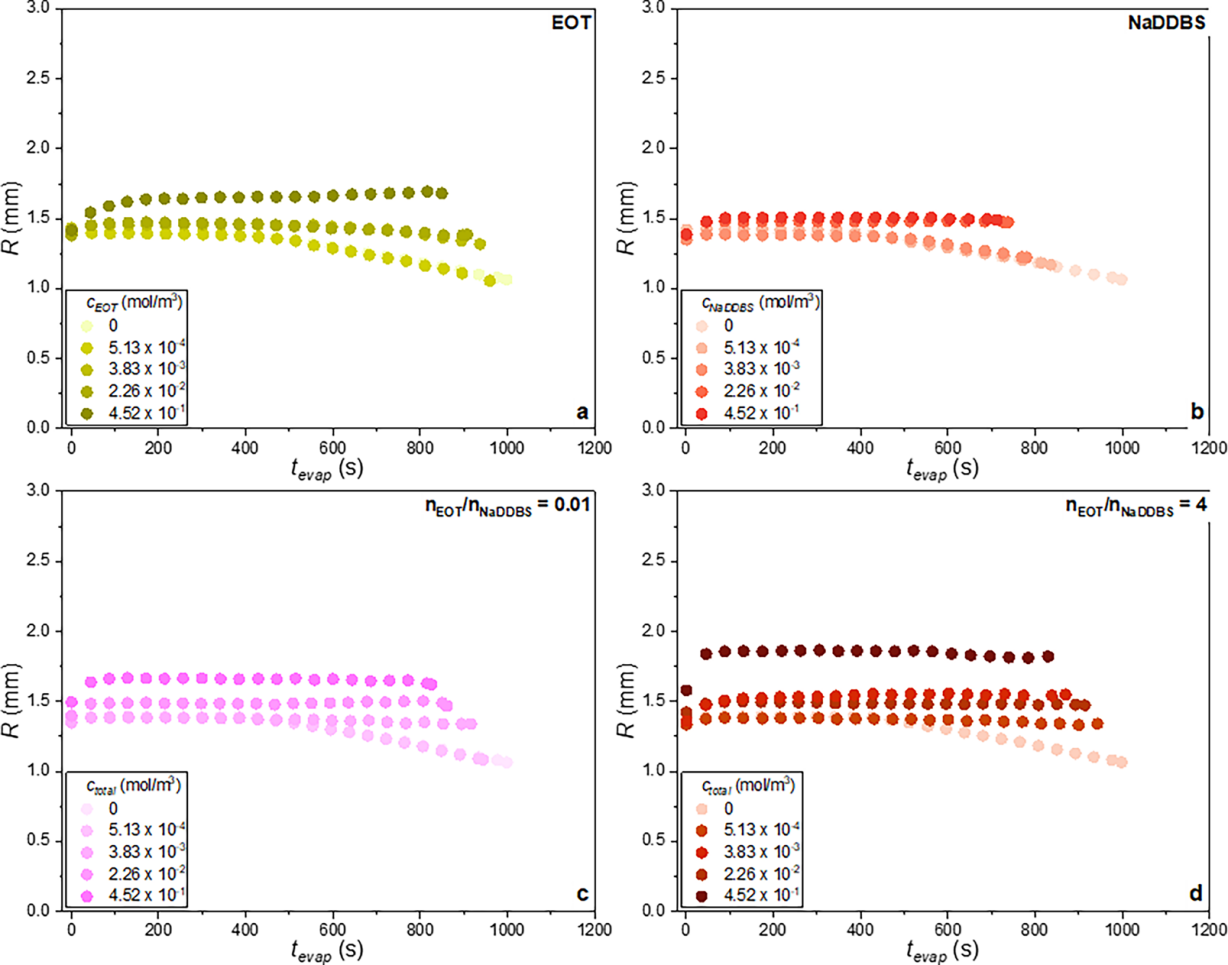
Evolution of the contact radius for the (a and b) single and (c and d) mixed surfactant-laden droplets.

As one can see in panels a and b of Figure 10 for the nonionic (EOT) and anionic (NaDDBS) surfactant cases and for all of the concentrations studied, when a droplet is deposited on the silane-coated glass slides, at *t_evap_* = 0 s, the initial static contact angle varies within a range of 66° ≤ *θ* ≤ 70°. Afterward, for the highest concentrations studied (*c* = 2.26 × 10^–2^ mol/m^3^ and *c* = 4.52 × 10^–1^ mol/m^3^) between *t*_evap_ = 0 s and *t*_evap_ ∼ 45 s a rapid decrease in the contact angle occurs, while the base radius increases (Figure 11a,b). In the literature,^15^ this is termed the spreading stage. In our study, this stage lasts for Δ*t*_evap_ ∼ 45 s, and the associated percentage of volume decrease is nearly 3.5 ± 0.4% and 3.8 ± 0.1% for single EOT and NaDDBS cases, respectively, suggesting that evaporation during the spreading stage also occurs but to a lesser extent.

The spreading stage is also observed for the mixed surfactant systems. As one can see for the *n*_EOT_/*n*_NaDDBS_ = 0.01 and 4 mixed droplets with *c*_total_ = 2.26 × 10^–2^ mol/m^3^ or *c*_total_ = 4.52 × 10^–1^ mol/m^3^, between *t*_evap_ = 0 s and *t*_evap_ ∼ 45 s, the contact angle rapidly decreases (Figure 10c,d) and the contact radius slightly increases (Figure 11c,d), corresponding to the spreading stage. Similar observations are made for the *n*_EOT_/*n*_NaDDBS_ = 0.1 and 1 mixed cases, as shown in Figures S9 and S10. The percentage of the droplet volume change during this time interval is small and approximately 3.1 ± 0.4%, 3.1 ± 0.1%, 3.6 ± 0.1%, and 3.1 ± 0.3% for the *n*_EOT_/*n*_NaDDBS_ = 0.01, 0.1, 1, and 4 mixed cases, respectively, which is consistent with literature observations.^57^ Although mixing surfactants can impact droplet spreading through synergistic effects, the concentration remains the predominant factor with higher concentrations promoting spreading. After the spreading stage, droplets evaporate under the CCR mode.

### CCR and CCA Modes of Evaporation

As one can see in Figure 12, the evaporation of pure DI water droplets takes place under the CCR and CCA modes. The same happens in the presence of the single EOT- and NaDDBS-laden droplets (Figure 12a,b), at low surfactant concentrations. At high surfactant concentrations, evaporation follows only the CCR mode because of the pinning of the contact line. For the surfactant mixtures, at *n*_EOT_/*n*_NaDDBS_ = 0.01 (Figure 12c) and *c*_total_ = 5.13 × 10^–4^ mol/m^3^, both CCR and CCA modes of evaporation are present, whereas for *n*_EOT_/*n*_NaDDBS_ = 4 (Figure 12d), droplets manifest only the CCR mode, even at low total initial surfactant concentrations. As we discussed in our previous work,^42^ higher EOT mole fractions in the mixtures cause stronger interactions among surfactant molecules, which may further promote the pinning of the three-phase contact line.

**Figure 12:**
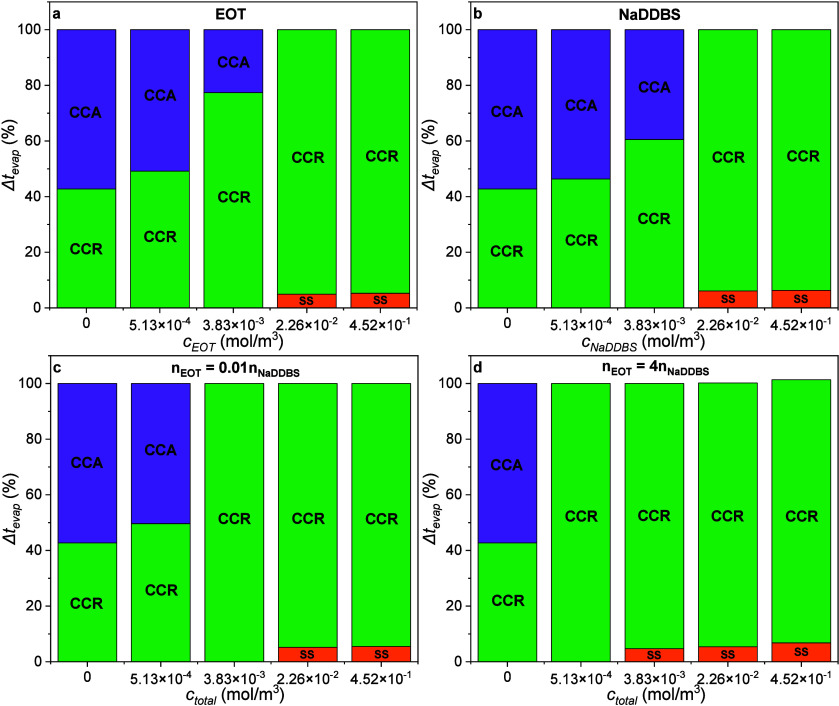
CCR and CCA modes of evaporation for the (a and b) single and (c and d) mixed surfactant-laden droplets. SS stands for the spreading stage.

For *n*_EOT_/*n*_NaDDBS_ = 0.1 and 1, the behavior is similar to the *n*_EOT_/*n*_NaDDBS_ = 0.01 and 4 cases, respectively (see Figure S11). In general, for the single EOT- or NaDDBS-laden droplets, as the total initial surfactant concentration increases, the duration of the CCA mode decreases, while for the surfactant mixtures, the CCA mode is only observed at mole ratios of *n*_EOT_/*n*_NaDDBS_ = 0.01 and 0.1 and when *c*_total_ = 5.13 × 10^–4^ mol/m^3^.

A few studies have considered pinning of the contact line during evaporation. For example, Kwieciński et al.^31^ studied the evaporation of SDS-laden aqueous droplets on hydrophobic surfaces and suggested that contact line pinning is induced by the formation of a disordered layer of surfactant aggregates with uneven wetting properties. However, as evaporation progresses and the surfactant concentration in the droplet increases, molecules might reorganize into a uniform layer, promoting even evaporation and contact radius retraction, as shown in panels b and c of Figure 4 for low total initial surfactant concentrations. Zang et al.^58^ mentioned that the CCR mode is usually observed, due to weak Marangoni flow, within droplets with *θ* < 90° on high-energy surfaces, and that surface roughness may also boost the pinning of the contact radius.

Kim et al.,^21^ in their study on the evaporation dynamics of water droplets on PMMA and PAMS hydrophobic substrates, suggested that during the CCR mode, surface roughness and chemical heterogeneities cause contact angle hysteresis, keeping the contact line pinned until the receding angle is reached. Hu and Larson^59^ focused on the vapor concentration distribution around pure water sessile droplets on hydrophilic glass slides and reported that an uneven evaporation can cause temperature variations along the droplet surface and create a surface tension gradient that could also contribute to contact line pinning.

### Evaporation Rate

The experimental droplet volume change over time, as calculated from the droplet profile following the procedure illustrated in the Supporting Information, was compared to the volume predicted by fitting the experimental data (*θ* and *R*) to the models of Picknett and Bexon,^44^ Rowan et al.,^45^ and Bourgés-Monnier and Shanahan^46^ to check their applicability for surfactant-laden systems. As one can see in Figures 13 and 14, for the pure DI water and EOT cases, respectively, the experimental volume decrease over time indicates a diffusion-controlled evaporation process that obeys eq 10.^25^ By a linear fit of the obtained volume data to the equation (*V*/*V*_max_)^2/3^ = *f*(*t*_evap_), the experimental evaporation rate (*ακ­(θ)_E_*) is acquired for the pure, single, and mixed surfactant systems, while by substituting the different *g­(θ)* approximations (eqs 5–7) in eq 10, evaporation rates *ακ­(θ)_P&B_*, *ακ­(θ)_R_*, and *ακ­(θ)_B&S_* can be calculated for the models of Picknett and Bexon,^44^ Rowan et al.,^45^ and Bourgés-Monnier and Shanahan,^46^ respectively.

**Figure 13:**
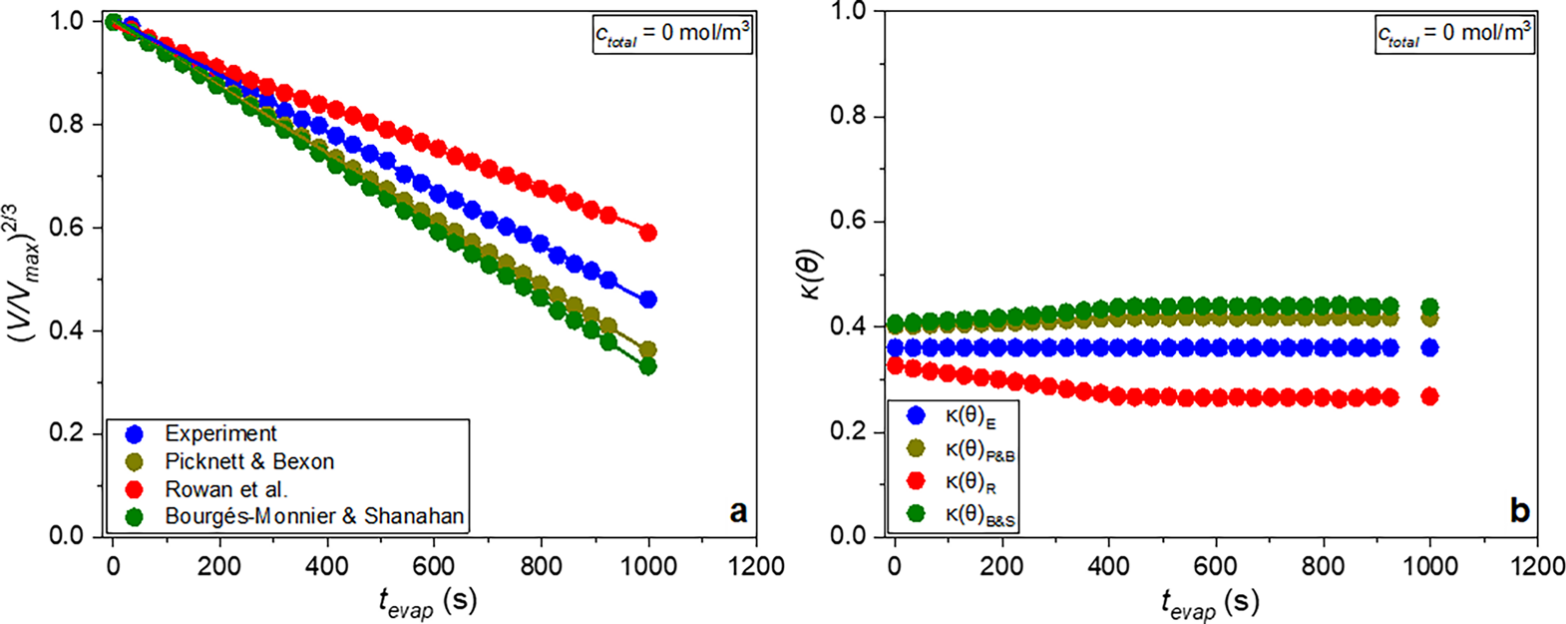
(a) Volume changes and (b) *κ­(θ)* values for the different models and pure DI water droplets. Solid lines represent the fitting of eq 10 to the experimental data for the different models.

**Figure 14:**
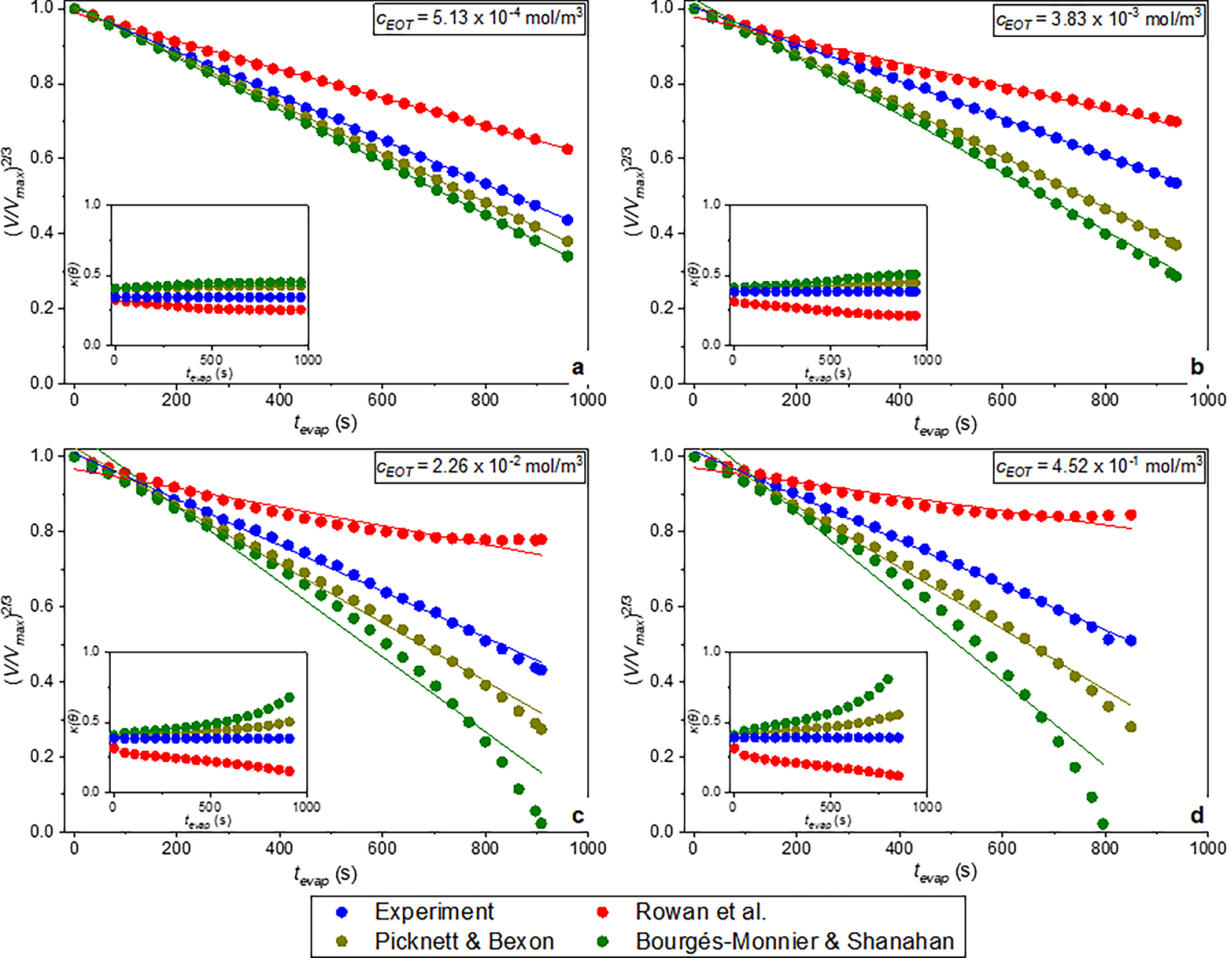
Volume changes for EOT-laden droplets at *c*_EOT_ = (a) 5.13 × 10^–4^, (b) 3.83 × 10^–3^, (c) 2.26 × 10^–2^, and (d) 4.52 × 10^–1^ mol/m^3^. Solid lines represent the fits of eq 10 to the experimental data for the different models. In the insets, *κ­(θ)* changes are presented.

These values are presented in Table 3 for surfactant concentrations that exhibit high regression coefficients (*R* ^2^ ≥ 0.99) based on the linear fitting of the predicted volume data. For the single EOT (Figure 14), this is true for *c* = 5.13 × 10^–4^ mol/m^3^ and *c* = 3.83 × 10^–3^ mol/m^3^. For high concentrations, the volume ((*V*/*V*_max_)^2/3^) decreases less linearly (*R* ^2^ < 0.97) with time; thus, the confidence of applying eq 10 to extract the evaporation rates, *ακ­(θ)*, decreases. This limitation is indicated via empty boxes in Table 3. For single NaDDBS and *n*_EOT_/*n*_NaDDBS_ = 0.01, 0.1, 1, and 4 mixtures, the behavior is similar to that of the EOT case, with (*V*/*V*_max_)^2/3^ = *f*(*t*_evap_) plots and α*κ­(θ)* values presented in Figures S12–S14 and Table S1. Relative percent errors in the evaporation rates between experiments and predictions are shown in Table S2 for cases in which *R* ^2^ ≥ 0.99.

For pure DI water (Figure 13a), all models deviate from the experimental data when *t*_evap_ ≥ 300 s. As shown in Table 4, the model of Bourgés-Monnier and Shanahan^46^ exhibits the highest deviation from the experimental results, while the models of Rowan et al.^45^ and Picknett and Bexon^44^ deviate the least, the values being within ±4.4%. However, the model of Picknett and Bexon^44^ also yields the closest time-resolved agreement with the experimental data, making it better able to describe the evaporation dynamics during the CCR and CCA modes.

**Table 4:** Absolute Percent Errors between Theoretical and Experimental EOT Evaporation Rates

EOT, Nonionic			
	absolute percent errors in *ακ(θ)*		
*c*_EOT_ (mol/m^3^)	P&B	R	B&S
0	29.4	25.0	33.8
5.13 × 10^–4^	23.6	24.7	32.6
3.83 × 10^–3^	16.6		33.2

From Figure 13b where the change in the *κ­(θ)* function is plotted for the different models, as calculated from eq 9, it becomes apparent that *κ­(θ)* is approximately constant during the CCR and CCA modes of evaporation. Up to *t*_evap_ = 426 s, there is a slight increase in the *κ­(θ)_P&B_* and *κ­(θ)_B&S_* values and a decrease in the *κ­(θ)* *_R_* value, marking the transition from the CCR to CCA mode (see Figure 10). At this time point, as shown in Figure 13a, the deviations of the different models from the experimental data become more pronounced, indicating that *κ­(θ)* variations are related to the evaporation modes and impact the predicted droplet volume behavior.

For the single EOT case with *c*_EOT_ = 5.13 × 10^–4^ mol/m^3^, surfactants appear to have a minor effect on the evaporation process, yielding results similar to those obtained for pure DI water. Relative errors in evaporation rates, shown in Table 4, are similar for the models of Picknett and Bexon^44^ and Rowan et al.^45^ (values within ±1.1%); however, the approach of Picknett and Bexon^44^ better predicts experiments, as shown in Figure 14a. At *c*_EOT_ = 3.83 × 10^–3^ mol/m^3^ (Figure 14b), the model of Picknett and Bexon^44^ remains consistent with eq 10, predicting a linear (*R* ^2^ = 0.99) volume decrease, while the model of Rowan et al.^45^ shows a reduced linearity with *R* ^2^ = 0.96. Compared also to the model of Bourgés-Monnier and Shanahan,^46^ the approach of Picknett and Bexon^44^ exhibits a smaller deviation from the experimental evaporation rate (see Table 4).

At *c*_EOT_ = 2.26 × 10^–2^ and 4.52 × 10^–1^ mol/m^3^, the deviation from the (*V*/*V*_max_)^2/3^ ∼ *t* law increases, as shown in panels c and d, respectively, of Figure 14, when the approximations of Rowan et al.^45^ or Bourgés-Monnier and Shanahan^46^ are applied to eq 10; thus, *ακ­(θ)_R_* and *ακ­(θ)_B&S_* values are not shown in Table 3. Even in these cases, the solution of Picknett and Bexon^44^ still follows the (*V*/*V*_max_)^2/3^ ∼ *t* law more closely, better describing experiments, but its deviation from the experimental data increases, e.g., to 36.5%, when *c*_EOT_ = 4.52 × 10^–1^ mol/m^3^. As observed from the subplots, constant *κ­(θ)* values yield a linear fit of eq 10 to the experimental volume data, whereas the fit deviates from linearity when *κ­(θ)* trends deviate from linearity, e.g., when *c*_EOT_ = 2.26 × 10^–2^ mol/m^3^ at *t*_evap_ ∼ 200 s for the solution of Rowan et al.^45^ and from *t*_evap_ ∼ 400 s for the solution of Bourgés-Monnier and Shanahan^46^ (see Figure 14c).

Overall, changes in the droplet volume depend on *κ­(θ)*. From the results summarized in Table 3, we can conclude that as the total initial surfactant concentration increases, the evaporation rate (*ακ­(θ)*) increases, as well, for all systems considered here. This increase in the evaporation rate with surfactant concentration, consistent with the decreased evaporation times shown in Figure 3, is mainly attributed to the dynamically changing droplet profile, presented in Figures 5 and 9, which leads to an increase in the area available for evaporation. Finally, our analysis suggests that the model proposed by Picknett and Bexon^44^ can better predict the experimental data, compared to the other models, for most cases and concentrations considered in this work.

This can be attributed to its versatility, as it can be applied to both the CCR and CCA modes of evaporation. The solution of Picknett and Bexon^44^ is considered precise for small droplets, modeled with a spherical cap, and masses ranging from 1 pg to above 40 mg in the case of organic droplets, as within this range the mean free path of the molecules is smaller than the dimensions of the droplet; thus, the evaporation is only diffusion controlled. The droplets in this study have a spherical cap geometry (see the demonstration in the Supporting Information) with volumes of approximately 3.3 μL or about 3.29 mg in terms of mass. In contrast, the solutions of Rowan et al.^45^ and Bourgés-Monnier and Shanahan^46^ are specifically designed for the CCR mode, with the model of Rowan et al.^45^ in our study being less accurate when *θ* < 55°.

Several factors may contribute to deviations between the fitting of eq 10 when the different models are applied to the actual experimental results. Equation 10 was derived under the ideal assumption that just above the droplet, the surface is saturated with vapor,^60,61^ which in practice is not the case. Additionally, since air typically contains some level of moisture, the assumption that at infinite distance the vapor concentration is zero is not entirely correct.

Furthermore, in surfactant systems, molecules can interfere with thermocapillary Marangoni convection, which generates flows in the bulk and accelerates the heat transfer from the solid substrate to the droplet surface, altering the surface temperature and, thus, the vapor concentration and diffusion rate.^62^ Especially for the surfactant mixtures and concentrations below the CMC, where equilibrium is reached in hours,^42^ the kinetics of surfactants (adsorption and desorption rates to and from surfaces) could also influence the contact angle, an effect that models do not consider. Moreover, the models have been developed for pure solvent droplets, and thus, surfactant interactions, or phase transitions due to the dynamically changing concentrations, are not taken into account. Finally, the thermal conductivity of the substrate can affect the heat transfer between the liquid–solid interface and influence evaporation.^63,64^

## Conclusions

In this work, the evaporation of surfactant-laden aqueous droplets was studied on slightly hydrophobic silane-coated glass slides. Two single surfactants, a nonionic one (EOT) and an anionic one (NaDDBS), both commonly encountered in the delivery of agrochemicals, were used, together with their mixtures at mole ratios *n*_EOT_/*n*_NaDDBS_ = 0.01, 0.1, 1, and 4. It was found that, compared to pure DI water droplets, the addition of surfactants accelerates evaporation, with an increase in the total initial surfactant concentration decreasing the evaporation time. The phenomenon was attributed to droplet flattening and the increase in the surface area, which favors evaporation. This effect appeared to be dominant at the majority of the surfactant concentrations tested, even though the surfactants accumulating at the interface would be expected to form barriers hindering evaporation. At low initial EOT concentrations, where the droplet profile resembled that of pure DI water, evaporation times were comparable for the surfactant-laden case. Interestingly, for NaDDBS at the same low concentrations, the droplet profile indicated a larger surface area, but evaporation times were significantly shorter compared to those of the pure DI water case. This was attributed to the disruption of hydrogen bonds at the interface, due to the surfactants, which would facilitate evaporation of the droplets.

When the evaporation of droplets containing single surfactants was compared, it was observed that the evaporation times of the EOT-laden droplets were higher than those of the NaDDBS ones. This observation took place even though EOT reduces the surface tension more effectively than NaDDBS and was attributed to the larger surface coverage provided by EOT compared to that of NaDDBS, which hindered water evaporation. In the case of surfactant mixtures, the evaporation times fell within the range obtained for the single surfactants. The measured evaporation times were closer to those measured for single EOT, even when the concentration of EOT was much lower than that of NaDDBS in the droplet, which was attributed to synergistic effects that result in higher surface EOT mole fractions.

For both single and mixed surfactant-laden droplets, an initial spreading stage was observed, at the highest concentrations studied, with a duration of a few seconds, followed by the constant contact radius mode. The duration of the constant contact radius mode was prolonged with higher initial surfactant concentrations. Additionally, for surfactant mixtures, it was noted that pinning of the contact line can be achieved at lower total initial concentrations compared to single surfactants, favoring a constant contact radius evaporation process.

Droplet evaporation rates showed that the approach of Picknett and Bexon^44^ yields the closest time-resolved agreement with experiments and accurately follows the (*V*/*V*_max_)^2/3^ ∼ *t* law, especially at high surfactant concentrations, where the models of Rowan et al.^45^ and Bourgés-Monnier and Shanahan^46^ failed to linearly fit the experimental volume data.

Our findings show that the addition of surfactants can promote spreading and evaporation of droplets on solid substrates. Combining nonionic and anionic surfactants can further improve the efficacy and application efficiency of agrochemicals.

## Supplementary Material



## Notes

Spherical cap geometry and droplet evaporation model analysis, structure of surfactant molecules, droplet volume estimation, quantification of surfactant adsorption on a solid substrate, GROMACS simulations, contact angle and contact radius temporal evolution, duration of the evaporation modes for mixed surfactant-laden droplets, and droplet volume temporal change and evaporation rates for single and mixed surfactant-laden droplets (PDF)
