# Supplementary material for: Effect of Surfactant Mixtures on the Evaporation Rate of Aqueous Sessile Droplets from Slightly Hydrophobic Substrates
Source: Langmuir. 2025 Sep 8;41(37):25774–88. doi: 10.1021/acs.langmuir.5c03712 (PMC12461931; doi:10.1021/acs.langmuir.5c03712)
Supplement: Supplementary file 1 [file la5c03712_si_001.pdf]

Supporting Information

Effect of Surfactant Mixtures on the Evaporation Rate of Aqueous Sessile Droplets from Slightly Hydrophobic Substrates

Kristo Kotsi[a], Teng Dong[a], Takeshi Kobayashi[b], Alexander Moriarty[b], Ian McRobbie[c], Alberto Striolo[d], Panagiota Angeli[*a]

[a]ThAMeS Multiphase, Department of Chemical Engineering, University College London, Torrington Place, London WC1E 7JE, U.K.

[b]Department of Chemical Engineering, University College London, Torrington Place, London WC1E 7JE, U.K.

[c]Innospec Ltd., Oil Sites Road, Ellesmere Port, Cheshire CH65 4EY, U.K.

[d]School of Sustainable Chemical, Biological and Materials Engineering, Sarkeys Energy Center, The University of Oklahoma, Norman, Oklahoma 73019, United States

[*]E-mail address: p.angeli@ucl.ac.uk

Table of Contents

Two-Dimensional Spherical Cap Geometry S4

Droplet Evaporation Models S5

Structure of Surfactant Molecules S6

Droplet Volume Estimation S6

Quantification of Surfactants Adsorption on a Solid Substrate S7

Indicative Calculation for Surfactants Adsorption on Droplet Base S8

Molecular Dynamics Simulations S10

Graphs S13

References S18

Table of Figures

Figure S1: Schematic of a droplet (modeled as a spherical cap) on a solid substrate. S4

Figure S2: (a), (b) NaDDBS molecule incorporating 12 carbon atoms in the non-polar tail and (c), (d) EOT molecule incorporating 16 ethoxylate groups in the polar head. S6

Figure S3: Volume of an EOT-laden droplet determined (a) with DSA100 and (b) numerically. S7

Figure S4: Volume for (a) DI water and (b) EOT-laden droplets determined using MATLAB® (red symbols) and implementing spherical cap geometry (gray symbols). S7

Figure S5: (a) Temporal evolution of the 5th overtone and (b) EOT, NaDDBS, surface excess for the bare and silanized quartz sensor. S9

Figure S6: Dissipation evolution over time for the 5th overtone of the bare and silanized quartz sensors as EOT and NaDDBS solutions are pumped through. S10

Figure S7: Layer formation in a (a) EOT- and (b) NaDDBS-laden droplet, acquired from the GROMACS simulations, at $\Gamma/\Gamma_{max_{EOT}} = 0.9$ and $\Gamma/\Gamma_{max_{NaDDBS}} = 1$, respectively. S12

Figure S8: *PDF* profiles of the EOT and NaDDBS surfactants against their distance from the simulation box center. S12

Figure S9: Evolution of contact angle for mixed surfactant-laden droplets. S13

Figure S10: Evolution of contact radius for mixed surfactant-laden droplets. S13

Figure S11: CCR and CCA modes of evaporation for the mixed surfactant-laden droplets. SS stands for spreading stage. S13

Figure S12: Volume changes of (a) NaDDBS and (b) $n_{EOT}/n_{NaDDBS} = 0.01$ cases, with solid lines fitting eq. **(10)** to experimental data for the different models. Subplots show $\kappa(\theta)$. ... S14

Figure S13: Volume changes of droplets with n_{EOT}/n_{NaDDBS} = (a) 0.1 and (b) 1, with solid lines fitting eq. **(10)** to experimental data for the different models. Subplots show $\kappa(\theta)$.....S15
Figure S14: Volume changes of $n_{EOT}/n_{NaDDBS} = 4$ case, with solid lines fitting eq. **(10)** to experimental data for the different models. Subplots show $\kappa(\theta)$..S16

Table S1: Evaporation Rate Data for Single and Mixed Surfactant-Laden Droplets……….S16
Table S2: Absolute Percent Errors between Theoretical and Experimental Evaporation Rates for Single and Mixed Surfactant-Laden Droplets..S18

Two-Dimensional Spherical Cap Geometry

For all the cases studied in the main manuscript, the initial droplet contact radius was found to be smaller than the calculated capillary length, and the Bond number was also below 1. This indicates that surface tension forces dominated the evaporation process, and allowed for the droplets to be modeled as spherical caps.[1,2]

The capillary length of a liquid is defined as:[3]

$$\boldsymbol{\lambda} = \sqrt{\frac{\boldsymbol{\sigma}}{\boldsymbol{\rho_l g}}} \quad \textbf{(S-1)}$$

where σ is the surface tension, ρ_l is the liquid density, and $g = 9.81\ \mathrm{m/s^2}$ is the gravitational acceleration. The Bond number is calculated as the ratio of gravitational over surface tension forces and is given by:[2]

$$\boldsymbol{Bo} = \frac{\boldsymbol{\rho_l g R^2}}{\boldsymbol{\sigma}} \quad \textbf{(S-2)}$$

where R is the droplet base contact radius.

If $Bo < 1$, the droplet maintains a spherical cap shape due to the dominance of surface tension. However, if $Bo > 1$, gravitational forces become significant, causing a droplet flattening.[4]

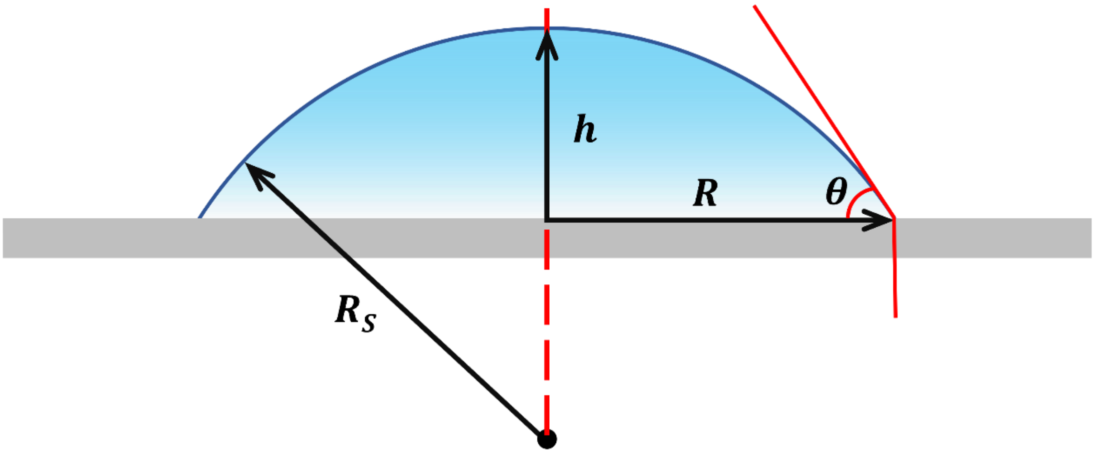

Figure S1: Schematic of a droplet (modeled as a spherical cap) on a solid substrate.

Three parameters are used to characterize spherical cap geometries: contact radius, height (h), and contact angle (θ). By geometry, the volume (V) of the spherical cap is correlated to the contact radius with the relationship:

$$\boldsymbol{R} = \left(\frac{\boldsymbol{3V}}{\boldsymbol{\pi\beta}}\right)^{\boldsymbol{1/3}} \boldsymbol{sin\theta} \quad \textbf{(S-3)}$$

where $\boldsymbol{\beta} = \mathbf{2} - \mathbf{3}\boldsymbol{cos\theta} + \boldsymbol{cos^3\theta}$ **(S-4)**

Droplet Evaporation Models

Picknett & Bexon[5] deposited methyl acetoacetate droplets on curved convex lens covered with a film of hydrophobic PTFE and studied both the constant contact radius and constant contact angle modes of evaporation. Instead of directly determining the evaporation rate of a micro-droplet, they evaluated the capacitance of the equiconvex lens formed by the droplet and its reflection on the substrate, whose size and shape were equivalent to those of the droplet. Using the analogy between diffusive flux and electrostatic potential, an exact solution for the volume decrease over time was developed, which can be expressed as:[5]

$$\frac{dV}{dt} = -\frac{2\pi D}{\rho_l}(c_{SC} - c_\infty)C \quad \textbf{(S-5)}$$

c_{SC} is the concentration of the water vapor around the droplet surface, c_∞ is the concentration of the vapor at infinite distance from the droplet surface, D is the diffusivity of the vapor to the surrounding air, and C is the capacitance of the equiconvex lens and is a function of the contact angle. To determine the capacitance, the researchers suggested two polynomial fits:[5]

for $0 \leq \theta < 0.175$ rad:

$$\frac{C}{R_S} = 0.63666\theta + 0.095910\theta^2 - 0.06144\theta^3 \quad \textbf{(S-6)}$$

or for $0.175 \leq \theta \leq \pi$ rad:

$$\frac{C}{R_S} = 0.00008957 + 0.6333\theta + 0.1160\theta^2 - 0.08878\theta^3 + 0.01033\theta^4 \quad \textbf{(S-7)}$$

where R_S is the radius of the sphere forming the spherical cap.

Rowan et al.[6] measured the height and contact angle of small water droplets on polymethyl methacrylate (PMMA) hydrophobic surfaces, during the CCR mode. They assumed a constant and radially outward concentration gradient, enabling a linear approximation when Fick's first law is applied. The researchers derived an approximate solution for the rate of volume decrease that reads as:[6]

$$\frac{dV}{dt} = -\frac{2\pi R_S D}{\rho_l}(c_{SC} - c_\infty)(1 - cos\theta) \quad \textbf{(S-8)}$$

Bourgés-Monnier & Shanahan[7] studied the evaporation of water and n-decane microdroplets on hydrophobic epoxy resin, polyethylene, and polytetrafluoroethylene surfaces, as well as on

hydrophilic glass surfaces, within the CCR mode. In their analysis, they considered a surface area around the droplet, where the vapor diffusion takes place, and for this area they defined a new system of coordinates sharing the same center with the center of the droplet. Assuming that the evaporation is radial, the following volume decrease approximation was obtained:[7]

$$\frac{dV}{dt} = \frac{2\pi R_S D}{\rho_l}(c_{SC} - c_\infty)\frac{cos\theta}{\ln(1-cos\theta)} \quad \textbf{(S-9)}$$

Structure of Surfactant Molecules

Representative structures of the NaDDBS and EOT surfactants are shown in **Figure S2**.

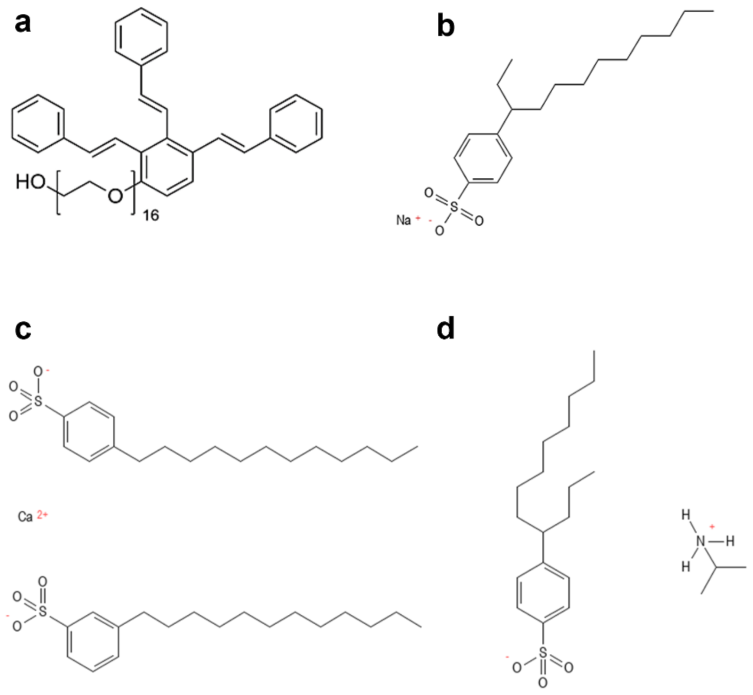

Figure S2: (a), (b) NaDDBS molecule incorporating 12 carbon atoms in the non-polar tail and (c), (d) EOT molecule incorporating 16 ethoxylate groups in the polar head.

Droplet Volume Estimation

As shown in **Figure S3**, the recorded droplet image (**Figure S3**a) is processed by converting it to grayscale and applying adaptive thresholding and then binarization to isolate the droplet (**Figure S3**b). The boundaries of the droplet are plotted, divided into horizontal slices (rows of pixels), and the droplet volume is calculated by summing the volumes of all the slices, each of them restricted by the droplet boundaries. Image processing and volume calculations were performed on MATLAB®.

In **Figure S4,** the theoretical volume points -plotted with those obtained from MATLAB®- are derived from eqs. **(S-3)** and **(S-4)**, using the contact angle and radius values directly measured with the DSA100 drop shape analyzer. The data confirm the MATLAB® calculations and indicate that the droplets maintain a spherical cap shape during the constant contact angle and constant contact radius modes. The slight irregularities in the theoretical line come from small fluctuations in the measurements, caused by image resolution and the instrument detection sensitivity at the droplet edges -variations that are typical of optical measurement methods.

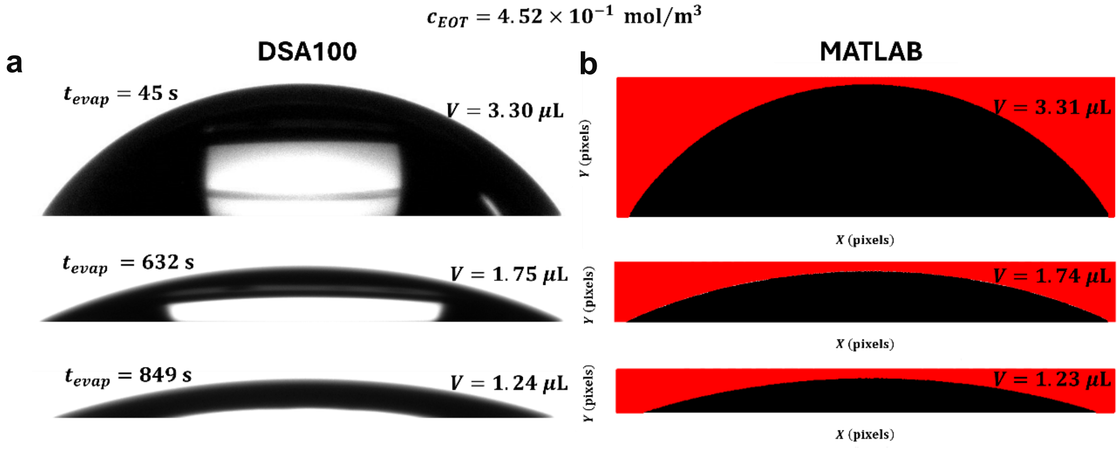

Figure S3: Volume of an EOT-laden droplet determined (a) with DSA100 and (b) numerically.

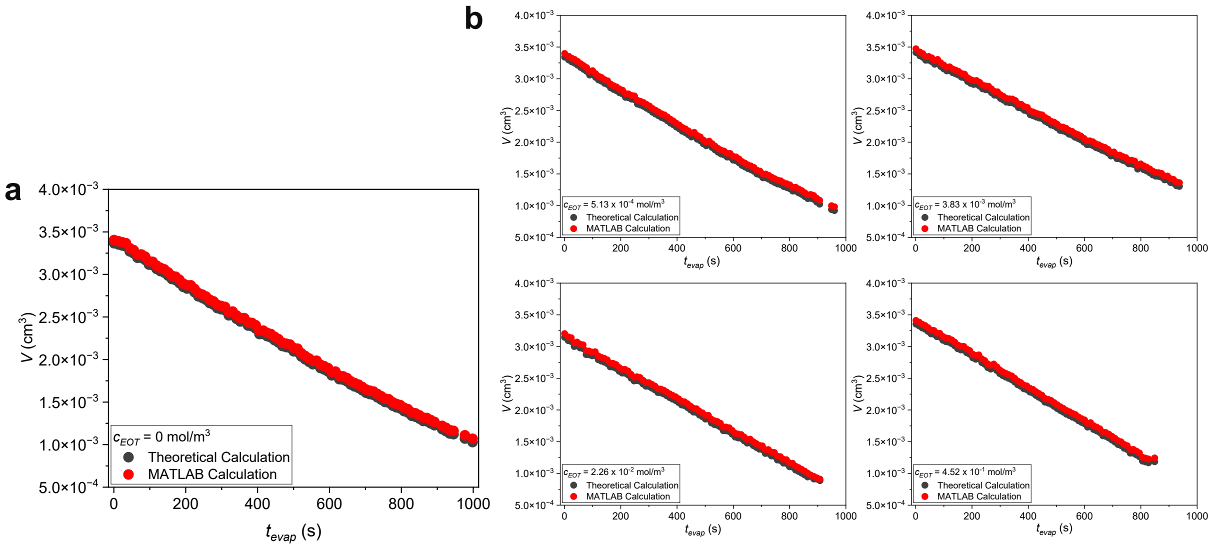

Figure S4: Volume for (a) DI water and (b) EOT-laden droplets determined using MATLAB® (red symbols) and implementing spherical cap geometry (gray symbols).

Quantification of Surfactants Adsorption on a Solid Substrate

A Q-Sense E4 quartz crystal microbalance (Biolin Scientific AB, Sweden) was used to determine the NaDDBS and EOT adsorption on coated silica sensors. Sensors were coated in a

vacuum chamber via silane vapor deposition, to match the surface coating of the solid substrates used for the evaporation studies. Measurements were performed at room temperature (~21°C), using the highest concentration from the evaporation studies, $4.52 \times 10^{-1}\ \mathrm{mol/m^3}$, to represent extreme adsorption conditions.

The silane coated sensor was carefully mounted in a flow module and ultrapure deionized water was initially pumped through the system until a smooth flow was established. After the baseline stabilized at 0 Hz, the surfactant solution was introduced in the flow module, and the change in frequency (Δf) over time was recorded, reflecting the mass (Δm) of surfactants adsorbed on the surface of the sensor. Representative results obtained are shown in **Figure S5**.

Quartz sensors have a 4.95 MHz nominal resonance frequency, which decreases as molecules adsorb onto their surface, following the Sauerbrey equation:[8]

$$\Delta m = -\frac{C \cdot \Delta f}{n} \qquad \textbf{(S-10)}$$

where n is the overtone number, and $C = 17.7 \frac{ng}{Hz \cdot cm^2}$ is the sensitivity constant of the quartz. Equation **(S-10)** assumes the formation of a thin rigid and uniform molecular film (dissipation below 2×10^{-6}).[9] The data analysis was performed for the 5th overtone, as this provides a balance between sensitivity and signal stability, with its dissipation lower than $9.1 \times 10^{-7} < 2 \times 10^{-6}$, as indicatively shown in **Figure S6**. A sample calculation is shown below.

At the end of the measurements the flow module, teflon tubing, and all other flow parts (except for the contact block with the electrode pins) were sonicated in a 2 wt % sodium dodecyl sulfate aqueous solution, followed by a deionized water rinse, and finally dried with nitrogen gas.

Indicative Calculation for Surfactants Adsorption on Droplet Base

Assuming a circular base for the droplet, its contact area with the substrate is calculated as:

$$A_{w-s} = \pi R^2 \qquad \textbf{(S-11)}$$

where R is the contact radius, obtained from the DSA100 instrument. The adsorbed surfactant moles (n_{w-s}) on the droplet substrate are calculated as:

$$n_{w-s} = A_{w-s} \cdot \Gamma_{max_{w-s}} \qquad \textbf{(S-12)}$$

where $\Gamma_{max_{w-s}}$ is the maximum surfactant concentration on the solid substrate as derived from the QCM data, when eq. **(S-10)** is applied at Δf_{max} (see **Figure S5**). In the plateau section, the

variations in Δf values are minimal, so we used the final point of the plateau as the equilibrium (Δf_{max}) value.

Indicatively, for $c_{EOT} = 4.52 \times 10^{-1}$ mol/m^3:

1) The initial total EOT moles in the droplet are: $n_{total} = 1.42 \times 10^{-9}$ moles.
2) From eq. **(S-11)**: $A_{w-s} = 6.25 \times 10^{-6}$ m^2.
3) From eq. **(S-10)** and $\Delta f_{max} = -21.6$ Hz: $\Delta_m = 7.64 \times 10^{-8}$ g/cm^2, and by dividing with the molecular weight of EOT ($MW_{EOT} = 1122$ g/mol) the maximum EOT concentration is calculated to be: $\Gamma_{max_{w-s}} = 6.81 \times 10^{-7}$ mol/m^2.
4) From eq. **(S-12)**: $n_{w-s} = 4.25 \times 10^{-12}$ moles.

This is the 0.3% of the initial total EOT moles in the droplet and is considered negligible.

The EOT moles adsorbed at the water-air interface are:

$$n_{w-a} = A_{w-a} \cdot \Gamma_{max_{w-a}} = 3.29 \times 10^{-11} \text{ moles} \quad (\text{or} \sim 2.3\% \ n_{total})$$

where $A_{w-a} = \pi(h^2 + R^2) = 8.73 \times 10^{-6}$ m^2, is the surface area of the droplet (modeled as a spherical cap, see above), and $\Gamma_{max_{w-a}}$ is the maximum surface excess at the water surface as determined in our previous work.[10]

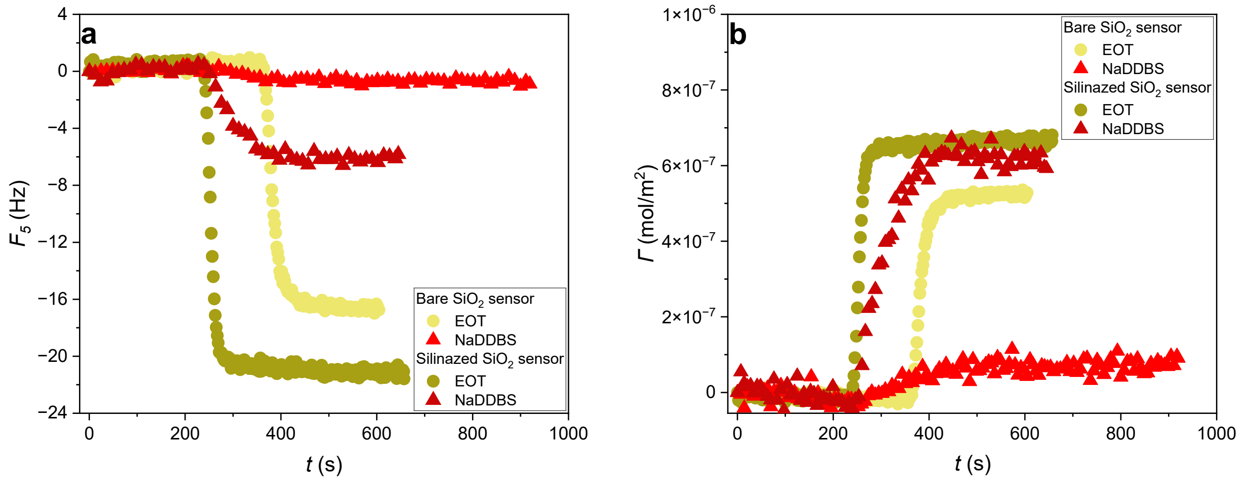

Figure S5: (a) Temporal evolution of the 5th overtone and (b) EOT, NaDDBS, surface excess for the bare and silanized quartz sensor.

Similarly, for NaDDBS at $c_{NaDDBS} = 4.52 \times 10^{-1}$ mol/m^3:

1) The initial total NaDDBS moles in the droplet are: $n_{total} = 1.42 \times 10^{-9}$ moles.
2) From eq. **(S-11)**: $A_{w-s} = 6.07 \times 10^{-6}$ m^2.

3) From eq. **(S-10)** and $\Delta f_{max} = -5.82$ Hz: $\Delta_m = 2.06 \times 10^{-8}$ g/cm^2, and by dividing with the NaDDBS molecular weight ($MW_{NaDDBS} = 348$ g/mol) its maximum excess is calculated to be: $\Gamma_{max_{w-s}} = 5.92 \times 10^{-7}$ mol/m^2.
4) From eq. **(S-12)**: $n_{w-s} = 3.60 \times 10^{-12}$ moles.

This is the 0.25% of the initial total NaDDBS moles in the droplet, negligible.

The NaDDBS moles adsorbed at the water-air interface are:

$$n_{w-a} = A_{w-a} \cdot \Gamma_{max_{w-a}} = 7.50 \times 10^{-11} \text{ moles} \;\; (\text{or} \sim 5.3\% \; n_{total})$$

where $A_{w-a} = \pi(h^2 + R^2) = 8.67 \times 10^{-6}$ m^2.

The calculations above assume that evaporation occurs through a series of quasi-equilibrium states, where EOT and NaDDBS rapidly adsorb at the water-air interface,[10] while evaporation rates are low (in the order of 10^{-5} cm^2/s, see **Table S1** and **Table 3** in the main manuscript).

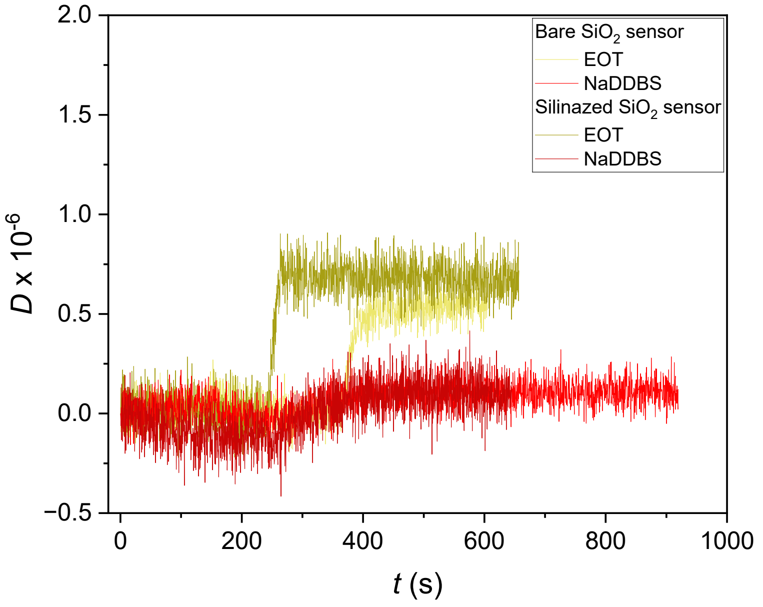

Figure S6: Dissipation evolution over time for the 5th overtone of the bare and silanized quartz sensors as EOT and NaDDBS solutions are pumped through.

Molecular Dynamics Simulations

Atomistic molecular dynamics simulations were performed using the software GROMACS 2021.5.[11–13] NaDDBS, EOT, and sodium ions were modeled using the OPLS/AA force field[14,15] while water was modeled with the SPC/E force-field.[16] Electrostatic interactions were treated using the Particle mesh Ewald (PME) method[17,18] (real-space cutoff: 1.2 nm; grid spacing: 0.16 nm; fourth-order interpolation scheme). Lennard-Jones interactions were truncated and

set to zero at 1.2 nm. The equations of motion were integrated using the leapfrog algorithm with an integration time step of 1.5 fs, while hydrogen-involving bonds were constrained with the LINCS algorithm.[19] The force-field validation was discussed in our previous work.[20] Aqueous EOT and NaDDBS solutions were simulated at surface concentrations (Γ): $0.0 \leq \Gamma \leq 4.6 \times 10^{-6}\ \mathrm{mol/m^2}$ -given that surfactant monolayers distort at $\Gamma_{EOT} {\sim} 2.0 \times 10^{-6}\ \mathrm{mol/m^2}$ and $\Gamma_{NaDDBS} {\sim} 4.5 \times 10^{-6}\ \mathrm{mol/m^2}$.

The simulations were carried out at constant room temperature, controlled by a velocity-rescaling thermostat[21] with a 0.1 ps coupling time constant. Initial configurations were generated with PACKMOL[22] in a $6 \times 6 \times 30\ \mathrm{nm^3}$ fixed box (**Figure S7**) ensuring an even surfactant distribution across both interfaces, as formed by periodic boundary conditions along the x, y, z directions. At low densities, random configurations led to surfactant aggregation, introducing bias in distributions. To mitigate this challenge, structured initial configurations were used to more accurately represent surfactant adsorption preferences and ensure their even distribution. After conjugate-gradient energy minimization, the systems were equilibrated for 10 ns under constant volume and temperature, followed by 120 ns production runs (80 million steps) under the same conditions.

To ensure that the reported surface water molecules and hydrogen bonds number represent equilibrium conditions, simulations were repeated twice for low surface concentrations (up to $1.1 \times 10^{-7}\ \mathrm{mol/m^2}$) and at least three times for higher surface concentrations, with less than 5% deviation between simulations. Given the slow dynamics of interfacial surfactants at high concentrations, initial 10 ns runs at 350 K were performed to accelerate equilibration, followed by 5 ns runs at room temperature.

Surface water molecules were identified using 0.2 nm radius probe spheres inserted from the vacuum into the surfactant solutions at 0.06 nm-spaced grid points on the xy-plane. To capture water molecules covered by surfactant tails, probes initially ignored surfactant molecules. After contact with water, the probe was retracted toward the vacuum, and if no other molecules were touched, the water was classified as surface water. Water molecules contacting surfactants and located more than 1 nm away, were also labelled as surface water.

Hydrogen bonds were identified using a custom Python script with a 0.32 nm distance and an 150° angle cutoff for surface water molecules. Hydrogen bonds between surface waters and between surface and non-surface water molecules were counted for each simulation frame, and the average hydrogen bonds per surface water molecule were calculated by accounting the

number of surface water molecules. Finally, the standard deviations for the number of surface water molecules and the hydrogen bonds per surface water molecule were calculated for the entire simulation trajectory. Representative snapshots for EOT- and NaDDBS-laden DI water systems (equilibrated conditions), as obtained from the GROMACS simulations, are shown in **Figures S7**a,b and their probability density function (*PDF*) against their distance from the center of the box (D_c) -indicative of their surface layer thickness- is presented in **Figure S8**.

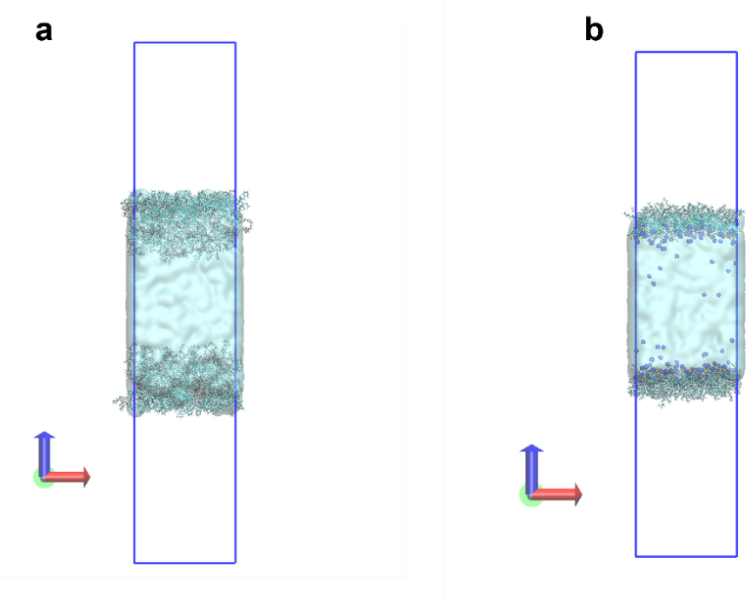

Figure S7: Layer formation in a (a) EOT- and (b) NaDDBS-laden droplet, acquired from the GROMACS simulations, at $\Gamma/\Gamma_{max_{EOT}} = 0.9$ and $\Gamma/\Gamma_{max_{NaDDBS}} = 1$, respectively.

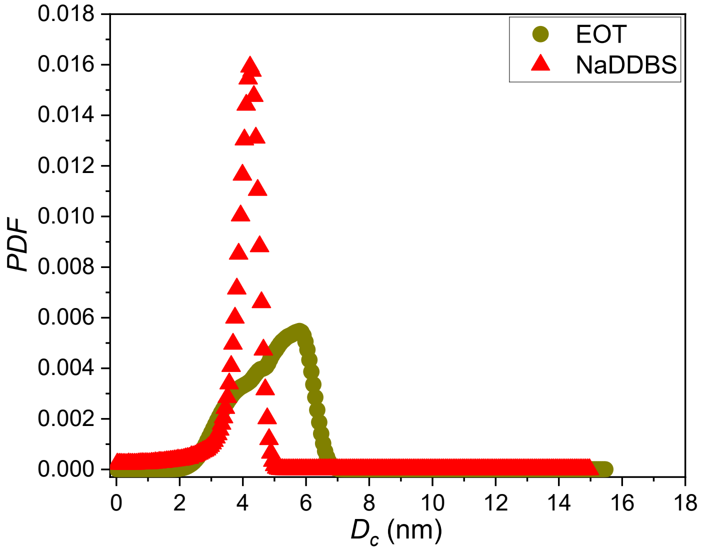

Figure S8: *PDF* profiles of the EOT and NaDDBS surfactants against their distance from the simulation box center.

Graphs

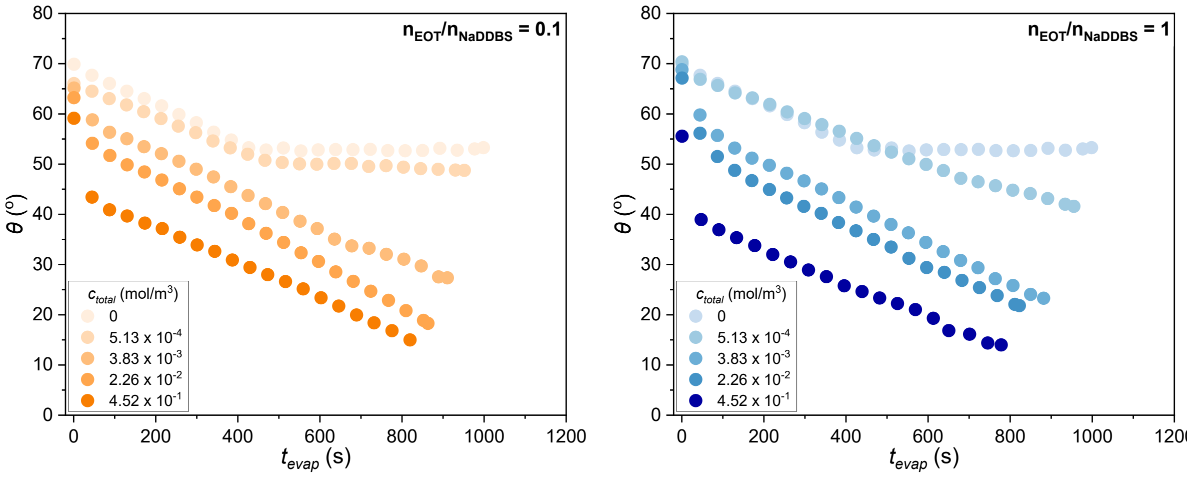

Figure S9: Evolution of contact angle for mixed surfactant-laden droplets.

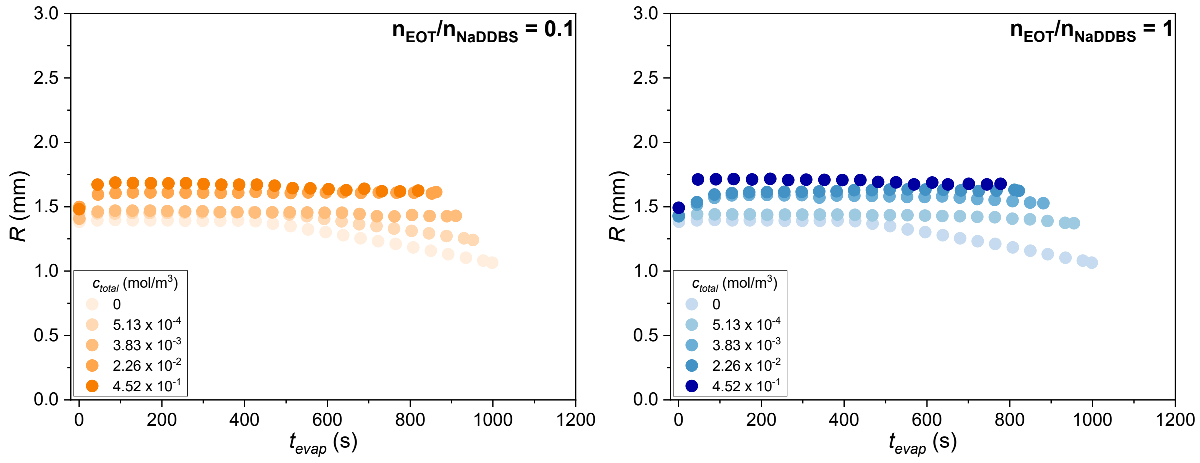

Figure S10: Evolution of contact radius for mixed surfactant-laden droplets.

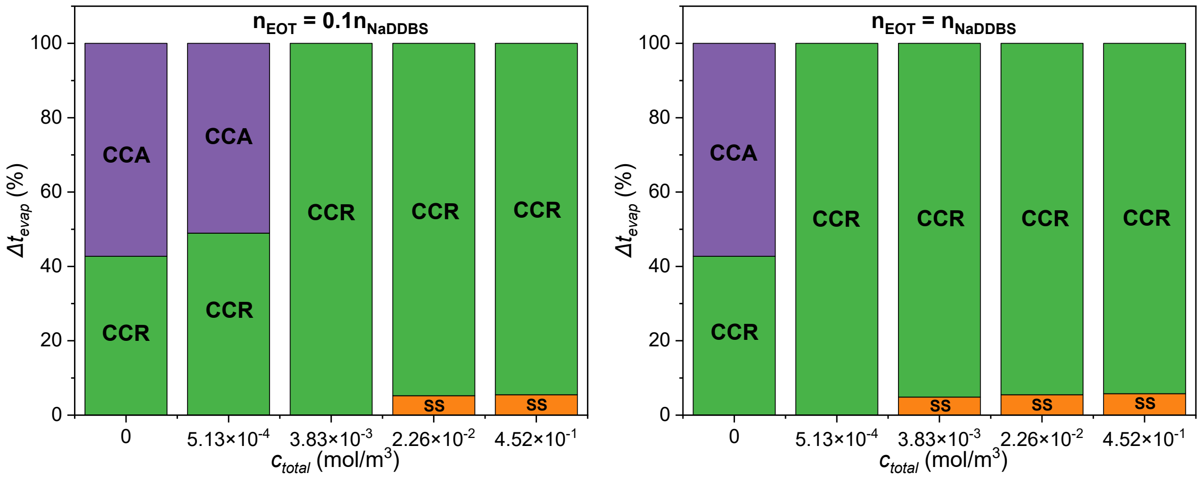

Figure S11: CCR and CCA modes of evaporation for the mixed surfactant-laden droplets. SS stands for spreading stage.

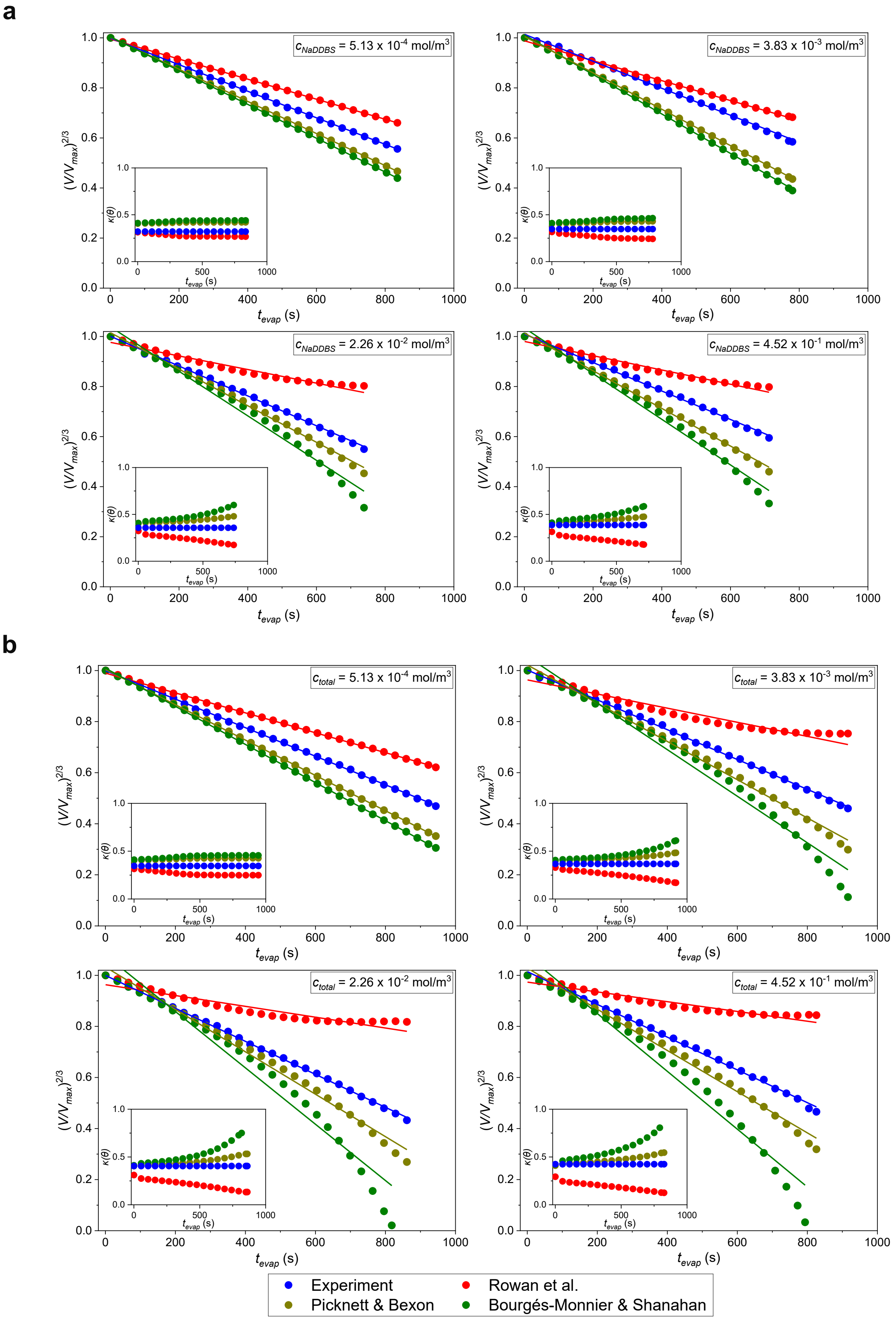

Figure S12: Volume changes of (a) NaDDBS and (b) $n_{EOT}/n_{NaDDBS} = 0.01$ cases, with solid lines fitting eq. **(10)** to experimental data for the different models. Subplots show $\kappa(\theta)$.

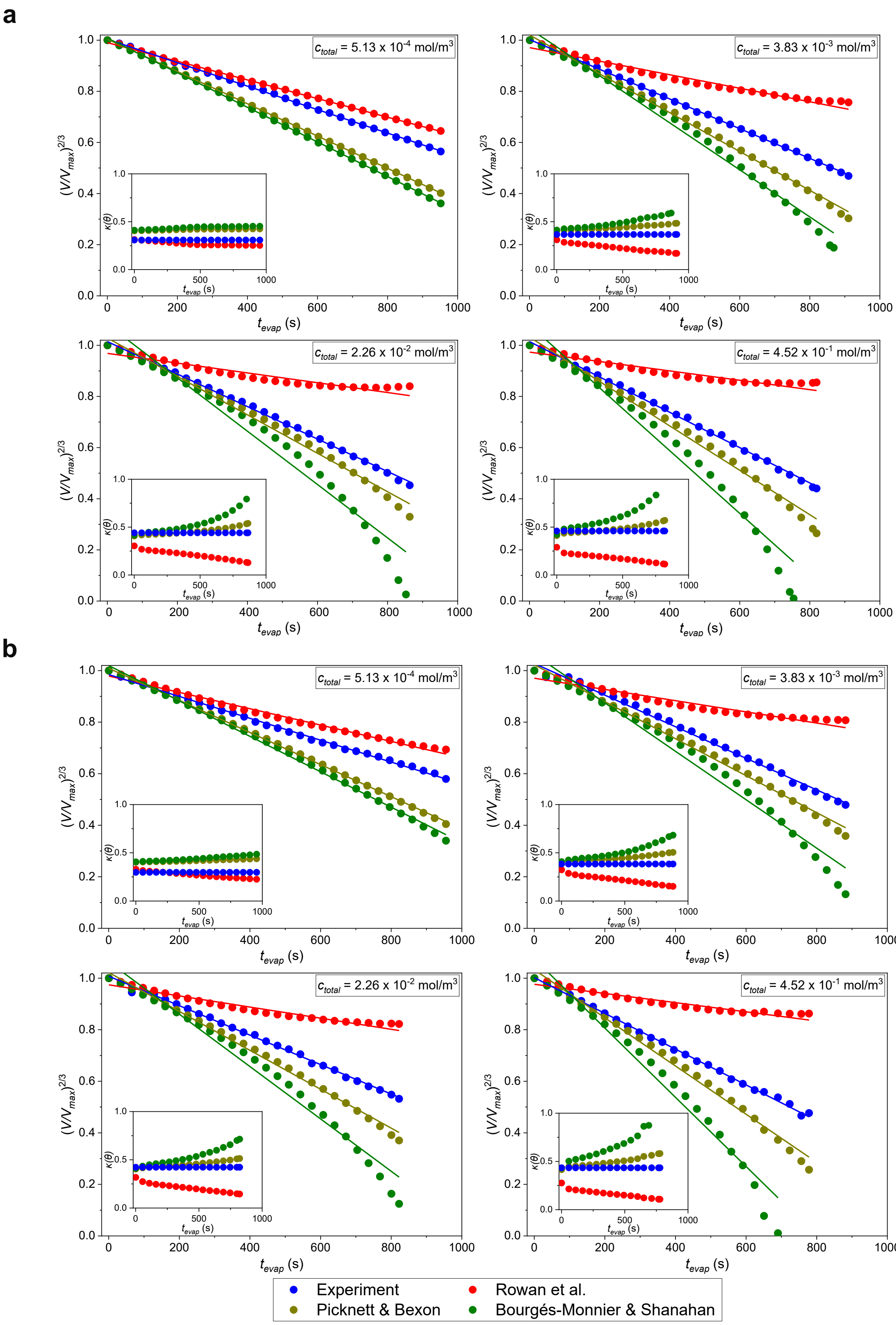

Figure S13: Volume changes of droplets with n_{EOT}/n_{NaDDBS} = (a) 0.1 and (b) 1, with solid lines fitting eq. **(10)** to experimental data for the different models. Subplots show $\kappa(\theta)$.

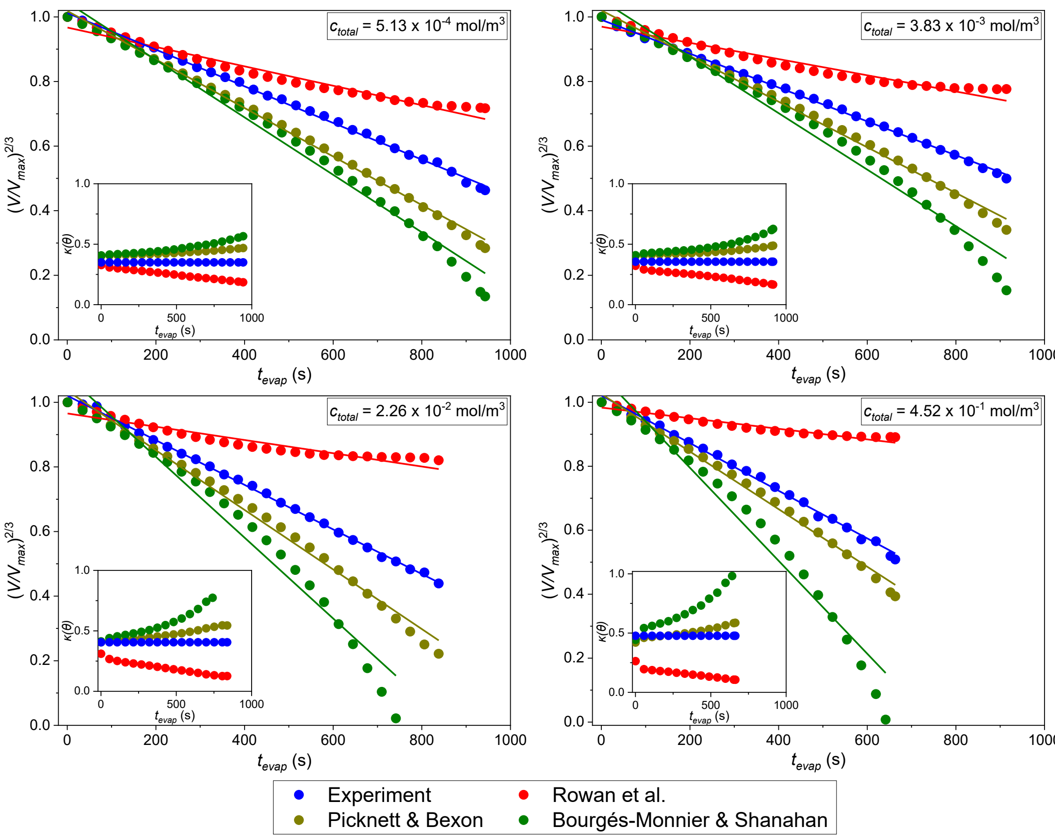

Figure S14: Volume changes of $n_{EOT}/n_{NaDDBS} = 4$ case, with solid lines fitting eq. **(10)** to experimental data for the different models. Subplots show $\kappa(\theta)$.

Table S1: Evaporation Rate Data for Single and Mixed Surfactant-Laden Droplets

c_{NaDDBS} (mol/m^3)	NaDDBS, Anionic			
	$a\kappa(\theta)$ (cm^2/s) $\times 10^{-5}$			
	$a\kappa(\theta)_E$	$a\kappa(\theta)_{P\&B}$	$a\kappa(\theta)_R$	$a\kappa(\theta)_{B\&S}$
5.13×10^{-4}	1.66	2.18	1.22	2.29
3.83×10^{-3}	1.80	2.23	1.36	2.42
2.26×10^{-2}	1.85	2.45		
4.52×10^{-1}	2.00	2.60		
c_{total} (mol/m^3)	$n_{EOT}/n_{NaDDBS} = 0.01$			
	$a\kappa(\theta)$ (cm^2/s) $\times 10^{-5}$			
	$a\kappa(\theta)_E$	$a\kappa(\theta)_{P\&B}$	$a\kappa(\theta)_R$	$a\kappa(\theta)_{B\&S}$
5.13×10^{-4}	1.79	2.25	1.25	2.40

$\mathbf{3.83 \times 10^{-3}}$	1.90	2.44		
$\mathbf{2.26 \times 10^{-2}}$	2.10	2.70		
$\mathbf{4.52 \times 10^{-1}}$	2.20	2.76		
c_{total} $(\mathrm{mol/m^3})$	$n_{EOT}/n_{NaDDBS} = 0.1$			
	$a\kappa(\theta)$ $(\mathrm{cm^2/s}) \times 10^{-5}$			
	$a\kappa(\theta)_E$	$a\kappa(\theta)_{P\&B}$	$a\kappa(\theta)_R$	$a\kappa(\theta)_{B\&S}$
$\mathbf{5.13 \times 10^{-4}}$	1.74	2.21	1.28	2.37
$\mathbf{3.83 \times 10^{-3}}$	1.90	2.49		
$\mathbf{2.26 \times 10^{-2}}$	2.28	2.70		
$\mathbf{4.52 \times 10^{-1}}$	2.38	2.86		
c_{total} $(\mathrm{mol/m^3})$	$n_{EOT}/n_{NaDDBS} = 1$			
	$a\kappa(\theta)$ $(\mathrm{cm^2/s}) \times 10^{-5}$			
	$a\kappa(\theta)_E$	$a\kappa(\theta)_{P\&B}$	$a\kappa(\theta)_R$	$a\kappa(\theta)_{B\&S}$
$\mathbf{5.13 \times 10^{-4}}$	1.64	2.07	1.29	2.50
$\mathbf{3.83 \times 10^{-3}}$	1.99	2.58		
$\mathbf{2.26 \times 10^{-2}}$	2.19	2.62		
$\mathbf{4.52 \times 10^{-1}}$	2.25	2.95		
c_{total} $(\mathrm{mol/m^3})$	$n_{EOT}/n_{NaDDBS} = 4$			
	$a\kappa(\theta)$ $(\mathrm{cm^2/s}) \times 10^{-5}$			
	$a\kappa(\theta)_E$	$a\kappa(\theta)_{P\&B}$	$a\kappa(\theta)_R$	$a\kappa(\theta)_{B\&S}$
$\mathbf{5.13 \times 10^{-4}}$	1.81	2.40		
$\mathbf{3.83 \times 10^{-3}}$	1.84	2.48		
$\mathbf{2.26 \times 10^{-2}}$	2.10	2.80		
$\mathbf{4.52 \times 10^{-1}}$	2.47	2.99		

Table S2: Absolute Percent Errors between Theoretical and Experimental Evaporation Rates for Single and Mixed Surfactant-Laden Droplets

c_{total} $(\mathbf{mol/m^3})$	**absolute percent errors in $\boldsymbol{\alpha\kappa(\theta)}$**		
	**NaDDBS, Anionic**		
	**P&B**	**R**	**B&S**
$\mathbf{5.13 \times 10^{-4}}$	31.3	26.5	38.0
$\mathbf{3.83 \times 10^{-3}}$	23.9	24.4	34.4
	$\boldsymbol{n_{EOT}/n_{NaDDBS} = 0.01}$		
$\mathbf{5.13 \times 10^{-4}}$	25.7	30.2	34.1
	$\boldsymbol{n_{EOT}/n_{NaDDBS} = 0.1}$		
$\mathbf{5.13 \times 10^{-4}}$	27.0	26.4	36.2
	$\boldsymbol{n_{EOT}/n_{NaDDBS} = 1}$		
$\mathbf{5.13 \times 10^{-4}}$	26.2	21.3	52.4

References

(1) Jenkins, A.; Wells, G. G.; Ledesma-Aguilar, R.; Orejon, D.; Armstrong, S.; McHale, G. Suppression of Crystallization in Saline Drop Evaporation on Pinning-Free Surfaces. *J. Chem. Phys.* **2023**, *158*, 124708.

(2) Shin, D. H.; Lee, S. H.; Jung, J. Y.; Yoo, J. Y. Evaporating Characteristics of Sessile Droplet on Hydrophobic and Hydrophilic Surfaces. *Microelectron. Eng.* **2009**, *86*, 1350–1353.

(3) Zang, D.; Tarafdar, S.; Tarasevich, Y. Y.; Dutta Choudhury, M.; Dutta, T. Evaporation of a Droplet: From Physics to Applications. *Phys. Rep.* **2019**, *804*, 1–56.

(4) Berthier, J. *The Physics of Droplets*; William Andrew Publishing, 2013.

(5) Picknett, R. G.; Bexon, R. The Evaporation of Sessile or Pendant Drops in Still Air. *J. Colloid Interface Sci.* **1977**, *61*, 336–350.

(6) Rowan, S. M.; Newton, M. I.; McHale, G. Evaporation of Microdroplets and the Wetting of Solid Surfaces. *J. Phys. Chem.* **1995**, *99*, 13268–13271.

(7) Bourgès-Monnier, C.; Shanahan, M. E. R. Influence of Evaporation on Contact Angle.

Langmuir **1995**, *11*, 2820–2829.

(8) Gutig, C.; Grady, B. P.; Striolo, A. Experimental Studies on the Adsorption of Two Surfactants on Solid-Aqueous Interfaces: Adsorption Isotherms and Kinetics. *Langmuir* **2008**, *24*, 4806–4816.

(9) Vogt, B. D.; Lin, E. K.; Wu, W. I.; White, C. C. Effect of Film Thickness on the Validity of the Sauerbrey Equation for Hydrated Polyelectrolyte Films. *J. Phys. Chem. B* **2004**, *108*, 12685–12690.

(10) Kotsi, K.; Dong, T.; Kobayashi, T.; Mc Robbie, I.; Striolo, A.; Angeli, P. Synergistic Effects between a Non-Ionic and an Anionic Surfactant on the Micellization Process and the Adsorption at Liquid/Air Surfaces †. *Soft Matter* **2024**, *20*, 523–534.

(11) Van Der Spoel, D.; Lindahl, E.; Hess, B.; Groenhof, G.; Mark, A. E.; Berendsen, H. J. C. GROMACS: Fast, Flexible, and Free. *J. Comput. Chem.* **2005**, *26*, 1701–1718.

(12) Pronk, S.; Páll, S.; Schulz, R.; Larsson, P.; Bjelkmar, P.; Apostolov, R.; Shirts, M. R.; Smith, J. C.; Kasson, P. M.; Van Der Spoel, D.; Hess, B.; Lindahl, E. GROMACS 4.5: A High-Throughput and Highly Parallel Open Source Molecular Simulation Toolkit. *Bioinformatics* **2013**, *29*, 845–854.

(13) Abraham, M. J.; Murtola, T.; Schulz, R.; Páll, S.; Smith, J. C.; Hess, B.; Lindahl, E. GROMACS: High Performance Molecular Simulations through Multi-Level Parallelism from Laptops to Supercomputers. *SoftwareX* **2015**, *1–2*, 19–25.

(14) Jorgensen, W. L.; Maxwell, D. S.; Tirado-Rives, J. Development and Testing of the OPLS All-Atom Force Field on Conformational Energetics and Properties of Organic Liquids. *J. Am. Chem. Soc.* **1996**, *118*, 11225–11236.

(15) Schweighofer, K. J.; Essmann, U.; Berkowitz, M. Simulation of Sodium Dodecyl Sulfate at the Water-Vapor and Water-Carbon Tetrachloride Interfaces at Low Surface Coverage. *J. Phys. Chem. B* **1997**, *101*, 3793–3799.

(16) Berendsen, H. J. C.; Grigera, J. R.; Straatsma, T. P. The Missing Term in Effective Pair Potentials. *J. Phys. Chem.* **1987**, *91*, 6269–6271.

(17) Darden, T.; York, D.; Pedersen, L. Particle Mesh Ewald: An Nlog(N) Method for Ewald Sums in Large Systems. *J. Chem. Phys.* **1993**, *98*, 10089–10092.

(18) Essmann, U.; Perera, L.; Berkowitz, M. L.; Darden, T.; Lee, H.; Pedersen, L. G. A Smooth Particle Mesh Ewald Method. *J. Chem. Phys.* **1995**, *103*, 8577–8593.

(19) Hess, B.; Bekker, H.; Berendsen, H. J. C.; Fraaije, J. G. E. M. LINCS: A Linear Constraint Solver for Molecular Simulations. *J. Comput. Chem.* **1997**, *18*, 1463–1472.

(20) Kobayashi, T.; Kotsi, K.; Dong, T.; McRobbie, I.; Moriarty, A.; Angeli, P.; Striolo, A. The Solvation of Na^+ Ions by Ethoxylate Moieties Enhances Adsorption of Sulfonate Surfactants at the Air-Water Interface. *J. Colloid Interface Sci.* **2025**, *682*, 924–933.

(21) Bussi, G.; Donadio, D.; Parrinello, M. Canonical Sampling through Velocity Rescaling. *J. Chem. Phys.* **2007**, *126*, 014101.

(22) Martinez, L.; Andrade, R.; Birgin, E. G.; Martínez, J. M. PACKMOL: A Package for Building Initial Configurations for Molecular Dynamics Simulations. *J. Comput. Chem.* **2009**, *30*, 2157–2164.
